# Current state of research on acupuncture for acne: a scoping review

**DOI:** 10.3389/fphys.2025.1661850

**Published:** 2025-10-03

**Authors:** Huiyuan Huang, Ying Liu, Shuhui Wu, Dan Zhao, Huie Zheng, Mingfang Zhu

**Affiliations:** ^1^ Department of Dermatology, The Second Hospital of Hunan University of Chinese Medicine, Changsha, Hunan, China; ^2^ Department of Rehabilitation and Health Care, Hunan Traditional Chinese Medical College, Zhuzhou, Hunan, China

**Keywords:** acupuncture, Acne vulgaris, scoping review, clinical research, Traditional Chinese medicine

## Abstract

**Objective:**

Acne vulgaris is recognized as one of the top eight most disabling dermatological diseases globally. Acupuncture has emerged as a clinically valuable and widely practiced intervention for acne, with the World Health Organization endorsing it as an effective non-pharmacological treatment. While existing evidence demonstrates acupuncture’s ability to significantly improve acne symptoms, the research remains scattered and lacks comprehensive synthesis. This scoping review systematically maps the current clinical research on acupuncture for acne treatment to identify knowledge gaps and inform future research directions.

**Methods:**

A systematic search was conducted across PubMed, EMBASE, the Cochrane Library, Web of Science, AMED, SinoMed, CNKI, WanFang, and VIP databases to identify relevant studies published between January 2014 and October 2024. Data extraction and synthesis were performed using descriptive statistics and visual analytics. The review followed the PRISMA-ScR guidelines and was prospectively registered with the OSF.

**Results:**

This study included 114 eligible studies, comprising 48 randomized controlled trials, 63 non-randomized interventional studies, and 3 systematic reviews, with the vast majority conducted in China. After 2019, the publication output of acupuncture studies for acne treatment showed a declining trend, which was generally consistent with changes in research funding. Cochrane risk-of-bias assessment revealed that the overall methodological quality of RCTs was moderate, with a low proportion of high-quality studies. The main acupuncture interventions for acne included filiform needle acupuncture, pricking-cupping, fire needling, autohemotherapy, bloodletting therapy, and catgut embedding at acupoints, with Ashi point (local lesion area) being the most frequently selected acupoint. Among the 16 outcome measures evaluated, the effective rate was the most commonly used indicator. Overall, acupuncture demonstrated good safety in treating acne, although fire needling showed a significantly higher frequency of adverse events compared to other therapies.

**Conclusion:**

As a globally prevalent complementary therapy, acupuncture has established a substantial research base for acne treatment; however, methodological limitations persist in existing studies. Future research should conduct multicenter, large-sample randomized controlled trials adhering to standardized reporting guidelines, develop comprehensive efficacy evaluation systems incorporating objective indicators, and investigate connections between clinical outcomes and mechanistic pathways. These efforts will elevate the evidence level for acupuncture in acne management.

**Systematic Review Registration:**

https://doi.org/10.17605/OSF.IO/S2QT6.

## 1 Introduction

Acne vulgaris is a common chronic inflammatory disorder of the pilosebaceous unit, clinically presenting with polymorphic lesions such as comedones, papules, pustules, and nodulocystic lesions (Vos et al., 2012). Epidemiological data indicate that acne affects approximately 9.4% of the global population, with a cumulative prevalence approaching 100% among adolescents. It ranks among the top eight dermatological conditions contributing to disability worldwide (Cruz et al., 2023; Saurat et al., 2024). While not life-threatening, acne often leads to significant sequelae including persistent erythema, post-inflammatory hyperpigmentation, and permanent scarring. These cutaneous manifestations profoundly impact patients’ psychosocial wellbeing, frequently correlating with anxiety disorders, clinical depression, and social withdrawal. Notably, severe cases may present with suicidal ideation, highlighting the condition’s mental health implications (Eichenfield et al., 2021). Recent global analyses reveal a sustained increase in the burden of acne among younger populations since 1990. In the United States alone, healthcare costs related to acne have reached approximately USD 3 billion (Zhu et al., 2025; Habeshian and Cohen, 2020). As one of the most burdensome dermatological conditions globally, acne management demands urgent clinical attention and optimized therapeutic strategies.

Current clinical management of acne vulgaris primarily involves the following approaches: pharmacological treatment (including topical and systemic medications), physical therapies (such as laser and photodynamic therapy), chemical peeling (e.g., glycolic acid and salicylic acid treatments), and dietary modifications (particularly restriction of high-glycemic and dairy products) (Mohsin et al., 2022; Reynolds et al., 2024). However, current therapeutic approaches are associated with notable limitations: Pharmacological treatments may induce drug resistance, while certain systemic medications (e.g., isotretinoin) carry teratogenic risks for women of childbearing potential (Albogami et al., 2024); Physical and chemical therapies frequently cause adverse effects including cutaneous dryness, irritation, and impaired skin barrier function; additionally, the efficacy of dietary interventions demonstrates significant interindividual variability (Mavranezouli et al., 2022). Given these clinical challenges, the development of safer and more effective complementary and alternative therapies has emerged as a priority in acne management. Acupuncture, a traditional external therapeutic modality in Chinese medicine, involves the stimulation of acupoints using various techniques and is valued for its simplicity, accessibility, affordability, and low incidence of adverse effects. Commonly used acupuncture methods for acne include filiform needle acupuncture, warm needle acupuncture, pricking and cupping therapy, and fire needle therapy (Mansu et al., 2018). Existing evidence suggests that acupuncture exerts therapeutic effects through multiple mechanisms, including modulation of immune function, suppression of inflammatory mediators, enhancement of local microcirculation, and regulation of sebum production (Chun-Yan et al., 2021). To date, acupuncture has been adopted in clinical practice across 196 countries and regions, with multiple techniques demonstrating promising efficacy in acne management (Jing et al., 2024; Ju, 2019; Zhao, 2021). The World Health Organization (WHO) officially recognizes acupuncture as an effective non-pharmacological therapeutic option for acne management (Duan et al., 2021).

However, due to the wide variety of acupuncture techniques with differing therapeutic advantages, there is currently a lack of comprehensive reports comparing different acupuncture therapies for acne treatment. Previous meta-analyses have primarily focused on quantitatively pooling the efficacy of acupuncture for acne. In contrast, scoping reviews represent a rigorous methodological approach for systematically mapping evidence, clarifying conceptual boundaries, and identifying research gaps within emerging fields. This scoping review aims to integrate existing evidence to clarify the therapeutic characteristics and appropriate clinical scenarios of various acupuncture modalities, thereby providing an evidence-based foundation for optimizing clinical decision-making in acupuncture therapy for acne, while simultaneously identifying critical directions for future research.

## 2 Methods

This scoping review was conducted in accordance with the Preferred Reporting Items for Systematic Reviews and Meta-Analyses extension for Scoping Reviews (PRISMA-ScR) guidelines. The study protocol was prospectively registered on the Open Science Framework (OSF; registration DOI: 10.17605/OSF.IO/S2QT6).

### 2.1 Identifying the research problem

A preliminary literature search indicated that acupuncture encompasses a wide range of therapeutic approaches and is widely applied in the clinical management of acne vulgaris. To gain a comprehensive understanding of the current research landscape regarding acupuncture interventions for acne, the research team developed the following guiding questions: (1) What are the main characteristics of existing clinical studies on acupuncture for acne? (2) What are the primary acupuncture treatment protocols being employed? (3) What is the methodological quality of the current evidence supporting the use of acupuncture for acne? (4) What outcome indicators have been used to evaluate treatment effectiveness and safety?

### 2.2 Search strategy

We systematically searched nine biomedical databases including PubMed, EMBASE, the Cochrane Library, Web of Science, the Allied and Complementary Medicine Database (AMED), the Chinese Biomedical Literature Database (CBM), China National Knowledge Infrastructure (CNKI), WanFang Database (WanFang), and the Chongqing VIP Chinese Science and Technology Periodical Database (VIP) from January 2014 to October 2024. The search strategy incorporated both Medical Subject Headings (MeSH) and free-text terms. For acne-related terms, we used: “acne vulgaris”, “acne”, and their corresponding Chinese equivalents. For acupuncture interventions, search terms included: “acupuncture”, “acupuncture therapy”, “electroacupuncture”, “fire needling”, “auricular acupuncture”, “acupoint injection”, “acupoint catgut embedding”, “autohemotherapy”, “bloodletting”, “warm needle moxibustion”, “plum-blossom needle”, and “moxibustion”, along with their Chinese translations. The search strategies were adapted for each database according to their specific indexing systems. Two reviewers independently screened the results, and any discrepancies were resolved through discussion. The complete search strategies for all databases are provided in Supplementary Appendix 1.

### 2.3 Inclusion and exclusion criteria

#### 2.3.1 Inclusion criteria

Studies were considered eligible if they met the following criteria: (1) Participants: Individuals clinically diagnosed with acne vulgaris; (2) Interventions: The intervention group received one or more forms of acupuncture therapy, including manual acupuncture, electroacupuncture, auricular acupuncture, fire needling, acupoint catgut embedding, autohemotherapy, acupoint injection, moxibustion, warm needle moxibustion, or plum-blossom needle therapy. The control group received treatments other than acupuncture; (3) Study design: Any type of clinical study, including randomized controlled trials (RCTs), non-randomized controlled trials (NRCTs), case reports, case series, cohort studies, cross-sectional studies, systematic reviews (SRs), and meta-analyses (MAs).

#### 2.3.2 Exclusion criteria

Studies were excluded based on the following criteria: interventions involving non-acupuncture therapies; animal or *in vitro* studies; studies with unavailable full-text; duplicate publications; non-systematic reviews; protocols; expert opinions; editorials; conference proceedings; and health bulletins.

### 2.4 Literature selection and data extraction

All identified citations were systematically managed using NoteExpress reference management software. Following a standardized protocol, two investigators (HHY and LY) independently screened the titles and abstracts of all records against the predetermined inclusion/exclusion criteria. Articles passing this initial screening phase underwent full-text evaluation for eligibility determination. Any discrepancies between reviewers were resolved through structured discussions, with unresolved cases being arbitrated by a senior third researcher (ZD). For all included studies, the following data were extracted using a pre-designed Excel form: (1) Basic study characteristics: first author, publication year, country/region, journal name, funding sources, publication language, and study design type; (2) For the studies’ contents: sample size, control group characteristics, acupuncture treatment protocol (including specific acupuncture modality, acupoint selection, needle specifications, treatment frequency and duration), primary outcome measures, and reported adverse events. To ensure data accuracy, dual independent extraction was performed with immediate cross-verification between investigators. Any identified inconsistencies were resolved through joint re-evaluation of the original publications.

### 2.5 Data analysis and reporting of results

The extracted study data were systematically organized in Excel spreadsheets for comprehensive data synthesis and descriptive analysis. We employed specialized software including GraphPad Prism 9.0 and ArcGIS Pro 3.2 for data integration, statistical analysis, and visualization. For RCTs, we conducted methodological quality and risk of bias assessments using Review Manager 5.4 software.

## 3 Results

### 3.1 Search results

**FIGURE 1:**
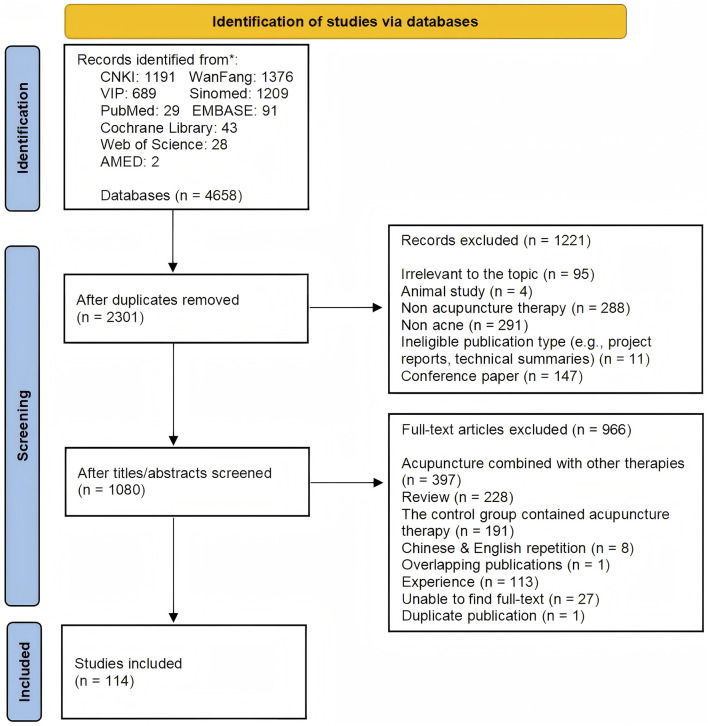
Study screening process.

Our systematic search identified 4,658 records, of which 2,357 were excluded as duplicates prior to screening. After excluding ineligible studies, this scoping review included 114 articles (see Supplementary Appendix 2 for Basic study characteristics). The literature search and selection process are detailed in Figure 1.

### 3.2 Research characteristics

**FIGURE 2:**
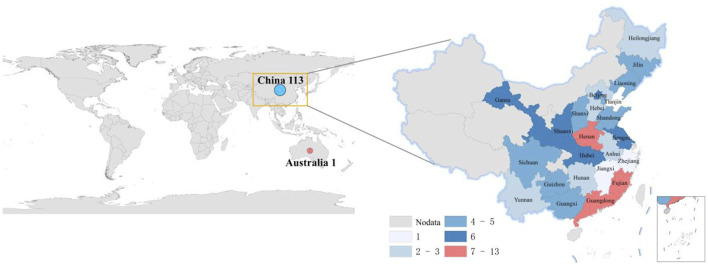
Geographic distribution of publications.

**FIGURE 3:**
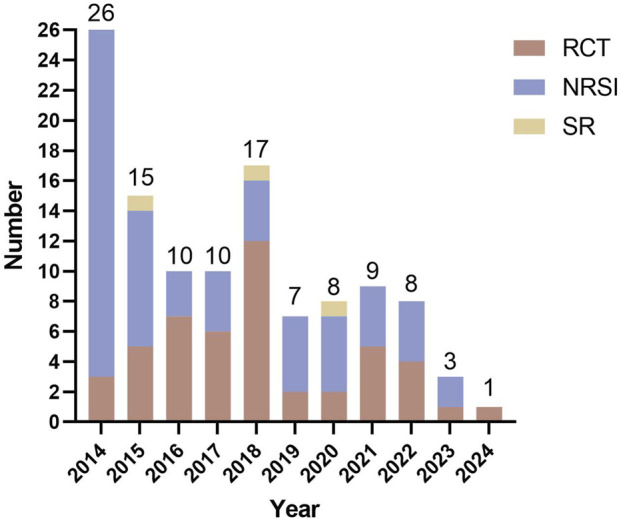
Publication counts by year and study type.

**FIGURE 4:**
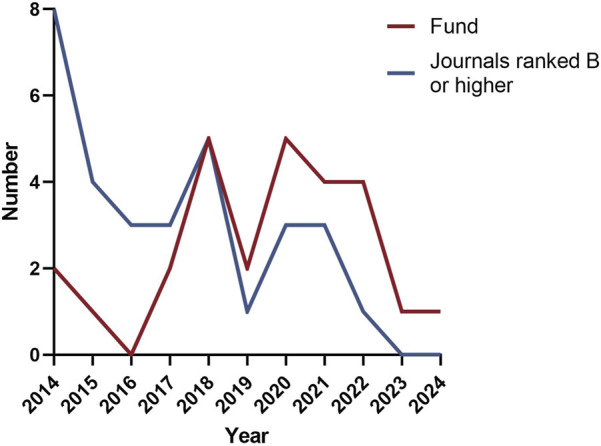
Annual publication trends of funding and high-quality journals.

This scoping review included 114 studies that were analyzed for their geographical distribution, publication timeline, funding status, and publication levels. The vast majority of studies (113 studies) originated from China, with only one study conducted in Australia. Within China, the highest research outputs came from the coastal provinces of Guangdong and Fujian, followed by Henan province in central China, while the remaining studies were distributed across other regions (see Figure 2 for geographical distribution). Analysis of publication years (Figure 3) showed all studies were published between 2014–2024, with 78 studies appearing before 2018 and a gradual annual decline thereafter. The included studies consisted of 48 RCTs, 63 non-randomized studies of interventions (NRSIs), and 3 SRs. The NRSIs comprised 17 NRCTs, 8 before-and-after controlled trials, 31 case series, 3 case reports, and 4 cohort studies. Twenty-seven studies reported funding sources, showing an overall increasing trend that peaked in 2020. By publication level, there were 5 SCI-indexed (Category A) studies, 26 CSCD/CSTPCD-indexed (Category B) studies, and 83 general publications (Category D). As illustrated in Figure 4, the publication trend in higher-tier journals (Category B and above) closely followed funding patterns, indicating that funded research was more likely to be published in more prestigious journals.

**FIGURE 5:**
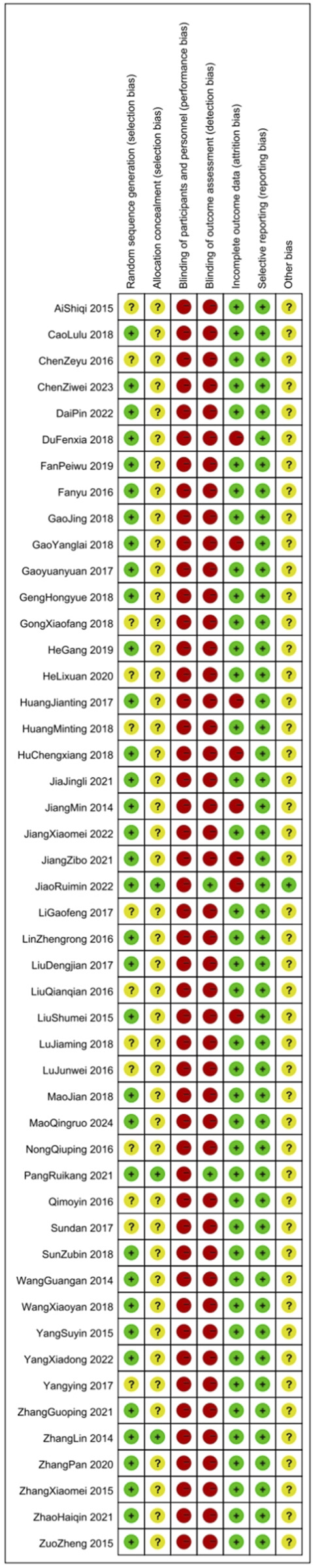
Risk of bias summary.

**FIGURE 6:**
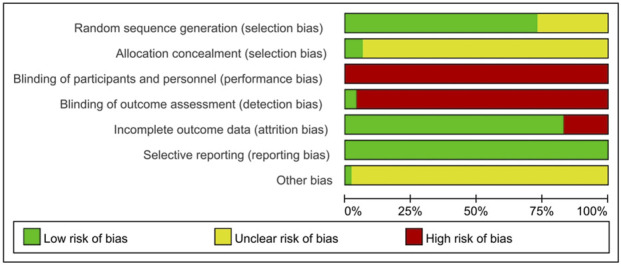
Risk of bias graph.

Through systematic synthesis of key study characteristics including authorship, publication year, study design, sample size, patient demographics, intervention protocols (for both treatment and control groups), and methodological features (such as randomization and blinding procedures), we systematically synthesized the available evidence (Supplementary Appendix 3). For RCTs, we conducted Cochrane risk-of-bias assessments using Review Manager 5.4 software (Figure 5). The results indicated moderate overall methodological quality among the evaluated RCTs, with only a minority demonstrating high-quality standards (Figure 6). Thirteen studies mentioned randomization without specifying the allocation method. Primary limitations included inadequate allocation concealment and blinding implementation - particularly challenging in acupuncture research due to inherent treatment characteristics (e.g., deqi sensation, acupoint specificity). Notably, two studies employing outcome assessor blinding were rated as low risk. While all 48 studies reported pre-specified outcome measures (scored as “low risk”), insufficient information about potential other biases led to “unclear” ratings for all but one study. For the non-randomized studies of interventions (NRSIs) included in this review, we evaluated the evidence according to the Oxford Centre for Evidence-Based Medicine (OCEBM): Levels of Evidence (2009) levels of evidence. These studies were primarily classified as level B or C, corresponding to moderate-to-low quality evidence. Therefore, the generalizability and reliability of their findings should be interpreted with caution.

### 3.3 Acupuncture treatment protocols

The 111 included clinical studies reported 47 distinct acupuncture intervention protocols. Based on the use of conventional needling techniques, these approaches were categorized into: conventional acupuncture therapy (filiform needle acupuncture) and specialized acupuncture techniques, the latter encompassing electroacupuncture, fire needling, plum-blossom needling, three-edged needling, and Fenggou needling. Furthermore, interventions were classified by complexity into single-modality acupuncture (one intervention method) and combined acupuncture therapy (two or more methods).

#### 3.3.1 Acupuncture therapy methods and acupoints selection

**FIGURE 7:**
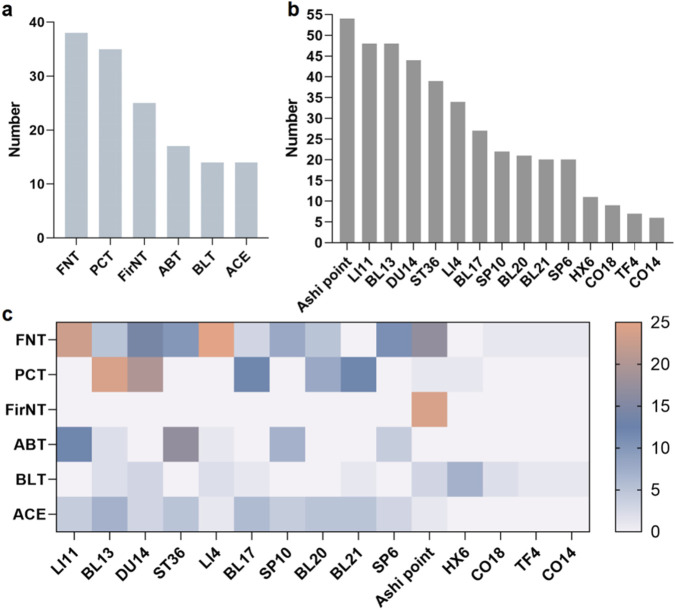
**(a)** Top 6 most frequently used acupuncture modalities for acne treatment; **(b)** Top 10 most frequently used conventional acupoints for acne treatment; **(c)** Usage frequency of common acupoints in different acupuncture modalities for acne treatment.

This study identified 47 distinct acupuncture treatment protocols involving 189 acupoints and 18 therapeutic approaches. The analysis demonstrated that conventional filiform needle acupuncture was the predominant intervention in current acne research, being used in 38 studies. Pricking-cupping therapy emerged as the most frequently combined modality, appearing in 35 studies. Specifically, six acupuncture techniques were employed in more than 10 studies: filiform needle therapy (n = 38), pricking-cupping (n = 35), fire needling (n = 25), autologous blood therapy (n = 17), bloodletting (n = 14), and acupoint catgut embedding (n = 14). The ten most frequently used conventional acupoints were: Quchi (LI11, n = 48), Feishu (BL13, n = 48), Dazhui (DU14, n = 44), Zusanli (ST36, n = 39), Hegu (LI4, n = 34), Geshu (BL17, n = 27), Xuehai (SP10, n = 22), Pishu (BL20, n = 21), Weishu (BL21, n = 20), and Sanyinjiao (SP6, n = 17). Beyond standard acupoints, Ashi points (n = 54) and auricular points - particularly Ear Apex (HX6, n = 11), Endocrine (CO18, n = 9), Shenmen (TF4, n = 7), and Lung (CO14, n = 6) - were among the most commonly selected special acupoints. We systematically analyzed the frequency of acupoint utilization across different acupuncture modalities for acne treatment. (Figure 7). Overall, filiform needle therapy and pricking-cupping therapy demonstrated the broadest clinical application, involving multiple high-frequency core acupoints. Ashi points showed the highest utilization rate in fire needle therapy, indicating that local stimulation of lesions represents the fundamental treatment strategy for acne with this modality. Autologous blood therapy predominantly selected immunomodulatory acupoints such as LI11 and ST36. Auricular acupuncture applications remained relatively limited, with the ear apex (HX6) being the most frequently used auricular point, primarily employed in bloodletting therapy.

#### 3.3.2 Parameters of filiform needle therapy

**FIGURE 8:**
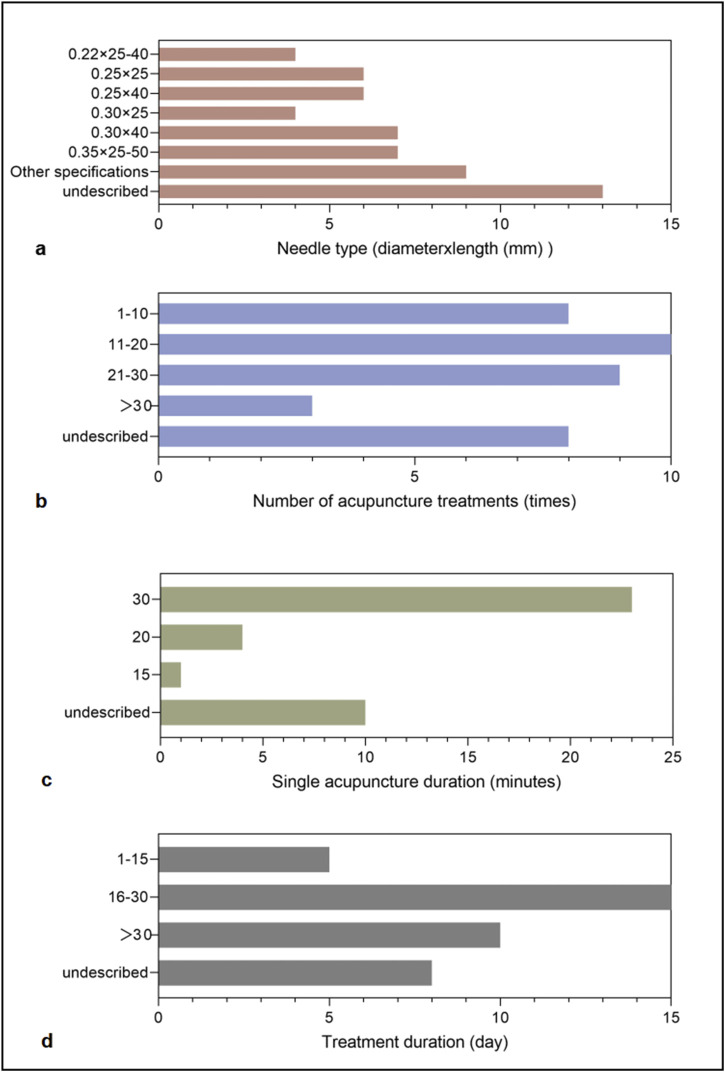
Technical parameters of filiform needle therapy. **(a)** Needle type (diameter × length, mm). **(b)** Number of acupuncture treatments (times). **(c)** Single acupuncture duration (minutes). **(d)** Treatment duration (days).

The dose-effect relationship of acupuncture stimulation is a critical factor influencing therapeutic outcomes, and filiform needle therapy was the most frequently employed modality in this study. Figure 8 summarizes the key parameters of filiform needle therapy for acne treatment, including needle specifications, total treatment sessions, needle retention duration per session, and overall treatment course. Among the 38 studies utilizing filiform needle therapy, 25 provided detailed needle dimensions (diameter × length), revealing that smaller-diameter needles produced less facial stimulation and were better tolerated by patients. A 30-min retention time was the most commonly adopted duration per session, with the majority of studies administering 11–30 treatment sessions over an approximately 30-day therapeutic course.

### 3.4 Outcomes

**FIGURE 9:**
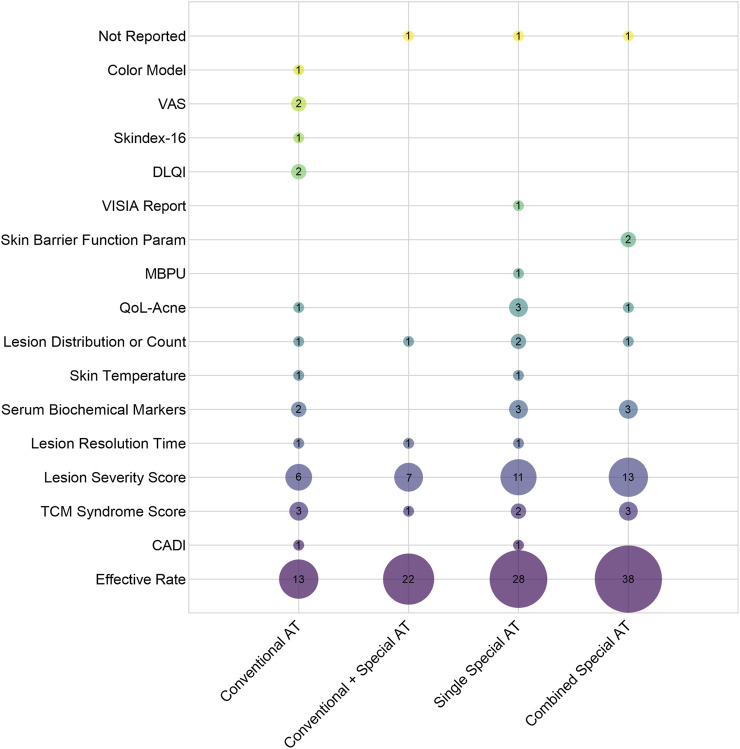
Bubble Chart of Outcome Measures in 111 Clinical Studies. (X-axis indicates intervention methods, and the Y-axis represents outcome indicators. The bubble size and the labeled values show the frequency of use of each indicator.). Note: AT: Acupuncture Therapies; CADI: Cardiff Acne Disability Index; TCM: Traditional Chinese Medicine; QoL-Acne: Quality of Life in Acne; MBPU: Microcirculatory Blood Perfusion Unit; DLQI: Dermatology Life Quality Index; Skindex-16: Skindex-16 scale; VAS: Visual Analogue Scale.

A total of 108 clinical studies incorporating 185 outcome measures were analyzed. These outcomes were systematically categorized into 16 distinct types: Effective Rate, Cardiff Acne Disability Index, Traditional Chinese Medicine Syndrome Score, Lesion Severity Score, Lesion Resolution Time, Serum Biochemical Markers including interleukin-2 (IL-2), interferon-gamma (IFN-γ), tumor necrosis factor-alpha (TNF-α), testosterone (T), and estradiol (E2), Skin Temperature, Lesion distribution or count, Quality of Life in Acne questionnaire, Microcirculatory Blood Perfusion Unit, Skin barrier function parameters, VISIA Skin Analysis Report, Dermatology Life Quality Index, Skindex-16 scale, Visual Analogue Scale, and Color Model parameters including RGB Area and Pigmentation Area. As shown in the bubble chart (Figure 9), the most frequently used outcome was Effective Rate (n = 101, 54.59%), followed by Lesion Severity Score (n = 37, 20.00%). Beyond these conventional measures, eight studies employed Serum Biochemical Markers to evaluate immunoinflammatory responses and hormonal profiles, specifically measuring IL-2, IFN-γ, TNF-α, T and E2 levels. Two studies documented Skin Temperature changes following acupuncture intervention, one study measured Microcirculatory Blood Perfusion Unit, and one study utilized Color Model analysis including RGB Area and Pigmentation Area as an innovative assessment approach.

### 3.5 Adverse events

**FIGURE 10:**
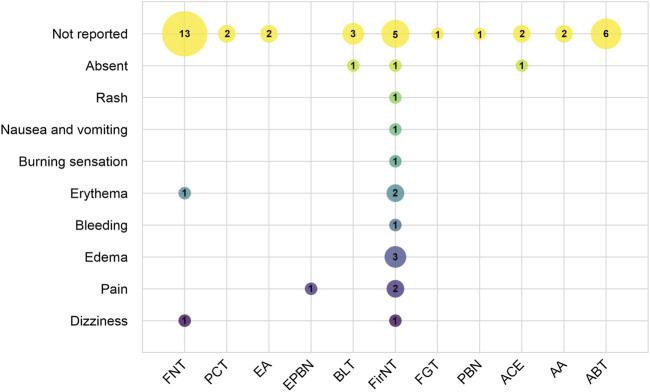
Distribution of Adverse Events. (X-axis indicates different acupuncture therapies, and the Y-axis represents adverse events. Bubble size and labels show the frequency of each adverse event.). Note: FNT: Filiform needling therapy; PCT: Pricking-cupping therapy; EA: Electroacupuncture therapy; EPBN: Electric plum blossom needle; BLT: Bloodletting therapy; FirNT: Fire needling therapy; FGT: Fenggou needling therapy; PBN: plum blossom needle; ACE: Acupoint catgut embedding; AA: Acupoint application; ABT: Autologous blood therapy.

Given the potential confounding effects arising from combined therapeutic interventions, which may interfere with accurate attribution of adverse events (AEs), this study specifically focused on adverse events reported in the 47 studies employing single acupuncture modalities. Among these, only 10 studies reported on AEs or complications, including 3 that explicitly stated no AEs occurred. The remaining 7 studies documented a total of 15 AE cases, with reported symptoms including dizziness, pain at needle insertion sites, local swelling, bleeding, erythema, burning sensations, nausea and vomiting, and rash. As shown in Figure 10, the recorded frequency of AEs across different acupuncture modalities indicated that fire needle therapy had a higher reporting frequency than other interventions, suggesting a relatively elevated risk of complications associated with this therapy. However, due to the lack of rigorous comparative statistical analysis, this observation reflects only an association rather than a causal relationship.

## 4 Discussion

This study provides a comprehensive scoping review of current clinical research on various acupuncture therapies for acne vulgaris, systematically evaluating both the breadth and depth of available evidence. Through detailed analysis of 114 included studies—encompassing study characteristics, treatment protocols, efficacy outcomes, and safety profiles—we found that while the majority of studies reported positive treatment effects, several critical limitations persist: inconsistent reporting standards, generally low levels of evidence, and overreliance on subjective outcome measures.

### 4.1 Characteristics of published studies and evidence quality

As a traditional therapeutic modality with over two millennia of clinical application, acupuncture has demonstrated efficacy in managing 532 documented disease conditions, exhibiting particular clinical value in dermatology for chronic inflammatory skin disorders including acne vulgaris (Jing et al., 2024; Hwang and Lio, 2021). However, current research output displays marked geographical imbalance and limited international engagement. Our scoping review identified 114 eligible studies, of which 113 originated from China with a single Australian publication, reflecting persistent challenges in acupuncture’s global integration. This study found that since 2019, both the volume of funding support and the number of related publications have shown a slight downward trend. This phenomenon may be partly attributable to the widespread disruption of research activities caused by the COVID-19 pandemic, and partly to our review’s inclusion criteria, which restricted analysis to acupuncture-only interventions and thus excluded a substantial number of contemporaneous trials involving combined acupuncture therapies. A systematic review (Cao et al., 2013) demonstrated that compared with pharmacotherapy alone, integrative treatment combining acupuncture with oral/topical medications significantly improves clinical cure rates in acne patients, suggesting this combined approach may represent an optimal clinical strategy. However, as the present study aims to systematically map the clinical research landscape of acupuncture monotherapy for acne, we exclusively included studies evaluating standalone acupuncture interventions to avoid potential confounding effects from combination therapies. Recent years have witnessed significant advancements in acupuncture research for acne treatment, as evidenced by increased growing publication outputs in high-impact journals. However, this field still faces challenges in generating high-level evidence. However, the current body of evidence still exhibits a structural imbalance, with NRSIs accounting for 55.3%, while higher-level evidence such as RCTs and SRs remains relatively insufficient. The NRSIs encompassed various designs including cohort studies, case-control studies, and before-after studies, whose substantial methodological heterogeneity not only creates structural unevenness in the evidence pyramid but also introduces considerable bias risks.

RCT are internationally recognized as the gold standard for evaluating clinical interventions, derive their evidential reliability from rigorous methodological quality. Our analysis identified significant shortcomings in key methodological domains: among included RCTs, deficiencies were observed in randomization procedures, allocation concealment, and blinding implementation. Randomization that is not adequately performed may lead to baseline imbalances; lack of allocation concealment can introduce selection bias; and insufficient blinding may give rise to expectation effects and measurement bias, all of which collectively contribute to systematic overestimation of treatment efficacy. Notably, in RCTs evaluating acupuncture interventions, blinding and allocation concealment present unique methodological challenges. First, acupuncturists, as intervention operators, must be aware of the treatment protocol, making blinding of practitioners inherently unfeasible. Second, although non-penetrating sham needles and other placebo measures can be employed to blind patients, differences in the sensation of “Deqi” often enable participants to discern between intervention and control groups, thereby introducing expectation effects. Furthermore, inadequate implementation of allocation concealment is also commonly reported. According to the evidence grading systems such as the GRADE framework (Guyatt et al., 2008), these methodological limitations generally confine such evidence to supporting only “weak recommendations,” rather than serving as a robust basis for clinical practice. As the birthplace of acupuncture, China possesses unique traditional expertise in this field. We therefore recommend prioritizing multicenter, large-scale RCTs that strictly adhere to international reporting standards for acupuncture interventions (STRICTA guidelines). Future study designs should pay particular attention to and innovatively address these methodological challenges. For example, implementing third-party assessment (blinding outcome evaluators) may compensate for the inherent impossibility of blinding acupuncturists as intervention providers, while strict application of centralized randomization procedures is essential to ensure adequate allocation concealment. Such rigorously conducted studies would significantly enhance both the evidence base and clinical translation potential of acupuncture therapy, facilitating its appropriate integration into global healthcare systems.

### 4.2 Acupuncture treatment protocols

Acupuncture therapy, as a comprehensive traditional Chinese medical technique that utilizes various needling methods to stimulate acupoints for disease prevention and treatment, has shown considerable potential in acne management. Through systematic analysis of 111 clinical studies, we have elucidated the diverse intervention protocols and clinical application patterns of acupuncture for acne treatment. The findings of this scoping review demonstrate that the most commonly employed acupuncture modalities include filiform needle acupuncture, pricking-blood cupping therapy, fire needling, autologous blood acupuncture, therapeutic bloodletting, and acupoint catgut embedding. The predominant acupoint selections were LI11, BL13, DU14, and Ashi points, among others. TCM theory attributes Quchi’s therapeutic effects to its dual capacity as the He-sea point of the Hand Yangming Large Intestine Meridian to simultaneously dispel wind-heat pathogens and purge internal fire. Contemporary studies demonstrate that needling LI11 and LI4 enhances mitochondrial ATP synthase activity and improves facial microcirculation, directly promoting acne lesion resolution (Zhou et al., 2019). Located on the posterior torso, DU14 and BL13 - the latter being a prototypical Back-Shu point - contain abundant sympathetic nerve terminals. These acupoints exert broad therapeutic effects on dermatological disorders by regulating corresponding internal organs through somatovisceral reflex mechanisms (Wang et al., 2016; Liu et al., 2019). Clinical evidence from Wang et al. (Wang JR. et al., 2021) confirms that pricking-cupping with bloodletting at DU14, BL13, and BL17 achieves 92.86% efficacy in reducing acne lesion area and hyperpigmentation. Ashi points, defined as dynamic reactive sites directly associated with pathological processes, are frequently selected as stimulation targets in clinical practice due to their characteristic presentation at acne lesion sites, including localized inflammation, tenderness, and pathological tissue changes (Son et al., 2010).

Therapeutic efficacy of acupuncture depends not only on acupoint selection but also critically on treatment dosage parameters, including needle specifications, needle retention time, treatment frequency, and the total number of treatment sessions. Our analysis reveals that 30 min represents the most commonly employed needle retention duration per session, with the majority of clinical protocols administering 11–30 treatment sessions over approximately 30 days. Research on the dose-effect relationship of acupuncture demonstrates that 30 min acupuncture stimulation can produce clinically meaningful effects in a range of disorders (Ye et al., 2021). Notably, studies demonstrate that acupuncture shows no statistically significant difference from sham acupuncture in treating moderate-to-severe acne within the first 4 weeks of treatment, suggesting that extending the therapeutic course beyond 12 weeks may enhance clinical efficacy (Jiao et al., 2022). Therefore, acupuncture parameters should be optimized based on individual patient characteristics, including symptom presentation, constitutional factors, and treatment response. We recommend future rigorously designed clinical trials to further investigate the dose-response relationship of acupuncture therapy for acne management.

### 4.3 Outcome measures

Among the 16 categories of outcome measures included in this study, the effective rate (ER) was the most frequently used indicator for evaluating the therapeutic effects of acupuncture on acne. However, substantial variability exists in how ER has been defined across studies, and most failed to provide explicit judgment criteria (see Supplementary Appendix 4 for details), making direct comparisons between results difficult and severely limiting cross-study comparability. A meta-analysis further highlighted that the current clinical trials on acne rely excessively on non-standardized efficacy evaluation systems, which may represent a key source of heterogeneity in the existing evidence base (Liu et al., 2022). The clinical assessment of acupuncture for acne involves multiple dimensions, including TCM syndrome differentiation, lesion characteristics, skin function, quality of life, and laboratory findings. Notably, lesion severity scores provide an intuitive reflection of clinical improvement. Current evidence (Bae et al., 2024) indicates that the most widely used acne severity assessment tools include the Pillsbury scale, Global Acne Grading System (GAGS), and Investigator’s Global Assessment (IGA), which aligns with the findings of our scoping review. The Pillsbury scale, as the first acne grading system, evaluates lesion type, count, and distribution, offering long-standing clinical utility. GAGS simplifies assessment by eliminating the need for exact lesion counting while maintaining high inter-rater reliability. IGA, valued for its reliability and clinical practicality, has become the predominant assessment tool in clinical trials. Additionally, quality of life measures including the Quality of Life in Acne (QoL-Acne), Dermatology Life Quality Index (DLQI), and Skindex-16 scale have been incorporated into outcome evaluation systems, enabling comprehensive assessment of acne’s impact on patients’ social functioning, psychological state, and overall quality of life. Of particular interest, our review identified several studies employing more objective assessment methods, including serum biomarkers (e.g., sex hormone levels, inflammatory cytokines) and quantitative skin parameters (e.g., facial coloration, blood perfusion, temperature). These quantitative measures enhance the objectivity and reliability of research outcomes, providing valuable insights for refining the efficacy evaluation framework for acupuncture in acne management. Therefore, this study recommends that future research prioritize the use of internationally recognized standardized assessment tools, such as the GAGS and IGA, and actively integrate objective biomarkers and physical parameters to establish a multidimensional efficacy evaluation system combining both subjective and objective measures, with the goal of generating more reliable and comparable evidence on treatment outcomes.

### 4.4 Safety profile and adverse events analysis of acupuncture for Acne treatment

To accurately assess the safety of acupuncture therapy for acne while minimizing confounding factors from combination therapies, this study focused specifically on analyzing the correlation between distinct single-modality acupuncture interventions and adverse events (AEs). Among the 47 included studies employing single acupuncture modalities, only 10 studies provided standardized AE reports involving techniques such as filiform needle acupuncture, electro-plum-blossom needling, and fire needling. The documented AEs primarily manifested as localized reactions (e.g., pain, edema, bleeding) and systemic responses (e.g., dizziness, nausea/vomiting). While acupuncture demonstrates an overall favorable safety profile, as an invasive stimulation technique, it may still induce minor adverse events (Bäumler et al., 2021; Huang et al., 2024). Filiform needle acupuncture, as the predominant intervention modality, achieves systemic regulation of qi and blood through specific acupoint combinations. Its adverse reactions (e.g., dizziness, localized erythema) may correlate with multiple factors including patient constitution, needle manipulation techniques, and treatment environment. Electro-plum-blossom needling combines electrical stimulation with plum-blossom needling’s therapeutic effects, enhancing local circulation and lymphatic drainage while potentiating anti-inflammatory responses (Zhang et al., 2014). Unlike filiform needle’s single-point stimulation, plum-blossom needling employs multi-point percussion to expand the treatment area, with electro-stimulation potentially intensifying discomfort. Notably, fire needling—which delivers combined mechanical and thermal stimulation—demonstrated higher AE frequency than other modalities, primarily causing localized tissue reactions (e.g., burning sensation, pain, erythema). This high frequency of occurrence may be related to procedural factors, such as needle temperature, duration of application, and operational precision, as well as patient-specific skin characteristics. To overcome the limitations of conventional fire-needle procedures, the electro-acupuncture fire-needle therapy has been developed (Wei et al., 2022), which employs digital control of needle temperature and insertion depth to standardize the intervention, thereby reducing the occurrence of adverse events and enhancing procedural reproducibility. Evidence suggests fire needling reduces inflammatory cytokine release and accelerates tissue repair through lesion stimulation (Xing et al., 2019), with most AEs resolving spontaneously within 30–60 min. In conclusion, acupuncture treatment for acne vulgaris generally exhibits a high safety profile; however, patients should be thoroughly informed of potential adverse reactions prior to treatment, and the technical training of practitioners should be strengthened. Given that the modern clinical mode of simultaneous physical and psychological treatment represents a mainstream approach, patient beliefs, treatment acceptance, and practitioner clinical experience may substantially influence therapeutic outcomes. Currently, qualitative data on these factors are lacking, and future research should systematically collect information on patient experiences, such as through semi-structured interviews or acupuncture sensation assessment scales, to provide a more comprehensive perspective for interpreting acupuncture efficacy.

### 4.5 Mechanistic discussion of various acupuncture therapies for Acne treatment

Acne vulgaris falls within the category of “Fei Feng Fen Ci” (肺风粉刺) in Traditional Chinese Medicine. TCM theory posits that the etiology and pathogenesis of acne are often related to external wind invasion and heat accumulation in the lung meridian; damp-heat accumulation and phlegm-dampness intermingling; toxic-heat accumulation with liver and kidney yin deficiency; and disharmony of the Chong and Ren meridians (Yang, 2019). Modern studies have demonstrated that acupuncture may exert anti-acne effects through multiple biological pathways. It can modulate the hypothalamic–pituitary–adrenal (HPA) axis to suppress androgen levels and sebum secretion, thereby reducing follicular occlusion and inhibiting the overgrowth of Cutibacterium acnes (Tan et al., 2009; Chang et al., 2023). At the same time, acupuncture can enhance immune function and downregulate inflammatory cytokines, promoting the resolution of inflammatory lesions such as papules and pustules and alleviating local erythema (Zuo et al., 2023). In addition, acupuncture improves local microcirculation and corrects abnormal follicular keratinization, thereby reducing comedone formation and facilitating lesion repair (Qu et al., 2017). Its regulatory effects on neurotransmitters and neuropeptides may further help relieve psychological symptoms frequently associated with acne, such as anxiety (Zeng et al., 2022). More recently, evidence has shown that acupuncture can restore gut–immune homeostasis by modulating the intestinal microbiota and promoting colonization of beneficial bacteria (Wang JH. et al., 2021). This finding provides a biomedical basis for classical Traditional Chinese Medicine (TCM) theories that “the lung and large intestine are interior–exteriorly related” and “the lung governs the skin,” thereby supporting the therapeutic concept of “simultaneous treatment of lung and intestine.” Taken together, these multi-target and multi-system mechanisms act synergistically to constitute the biological foundation of acupuncture therapy for acne and illuminate the modern scientific underpinnings of relevant TCM principles.

A clinical study demonstrated that filiform needle acupuncture at Chize (LU5), Fengmen (BL12), BL13, and LI4 acupoints combined with peri-lesional facial needling significantly upregulated serum levels of Natural Killer T cells (NKT), CD3^+^, and CD8^+^ T-cell subsets in acne patients. This immunomodulatory effect contributes to clinical improvement of acne lesions (Xi et al., 2024). Data mining analysis revealed acne vulgaris as the most frequent dermatological indication for bloodletting cupping therapy in clinical practice (Zhang et al., 2023). The therapeutic mechanism involves synergistic actions between the mechanical stimulation of three-edged needle puncturing and negative pressure from cupping, which enhances local circulation and lymphatic drainage while activating the Neuro-Endocrine-Immune (NEI) network - potentially representing the key pathway for its anti-acne effects (Xu et al., 2013; Chen et al., 2020). Evidence indicates that fire needling therapy exerts its therapeutic effects through multiple pathways. The localized thermal effect directly disrupts the survival microenvironment of Propionibacterium acnes while promoting leukocyte extravasation and enhancing phagocytic activity (Ma et al., 2024). These effects promote the clearance and absorption of inflammatory mediators, thereby maintaining homeostasis of the local immune microenvironment (Wang, 2018). Moreover, the thermal stimulation induced by fire needling causes vasodilation and alters vascular permeability at acne lesions, promoting the release of reparative factors including vascular endothelial growth factor (VEGF) to accelerate tissue repair processes (Song et al., 2024). Acupuncture stimulation of the concha region has been shown to activate the vagal cholinergic anti-inflammatory pathway (Ouyang et al., 2020). Animal studies further demonstrate that the combined therapy of auricular acupressure and ear apex bloodletting promotes M2-type macrophage polarization in the spleen of acne-model rats while significantly reducing expression levels of pro-inflammatory cytokines Tumor Necrosis Factor-α (TNF-α) and Interleukin-1 β (IL-1β). It is noteworthy that auricular acupressure may exert its anti-inflammatory and anti-acne effects by suppressing TLR2/NF-κB expression (Zuo et al., 2023). In conclusion, acupuncture therapy, characterized by a wide spectrum of techniques involving distinct acupoint prescriptions and instrument utilization, exerts positive effects on acne lesion resolution via multi-target and multi-pathway mechanisms. Notably, the various acupuncture modalities exhibit complementary profiles in terms of acupoint selection and therapeutic outcomes.

## 5 Limitations

As the first scoping review to systematically consolidate clinical evidence on acupuncture for acne treatment, this study addresses a critical gap in comprehensive synthesis within the field. However, several limitations should be acknowledged: Among the 114 included studies, 113 originated from China, and this imbalance restricts the generalizability of findings across different countries, regions, and cultural contexts. The literature search was restricted to Chinese and English databases due to language constraints, potentially omitting relevant studies in other languages and exacerbating language bias. Methodologically, while descriptive statistics effectively summarized frequently used acupuncture techniques and acupoints, they did not permit in-depth analysis of acupoint combination patterns or their clinical implications. Opportunities for improvement in data visualization remain, such as using matrix heatmaps or acupoint co-occurrence networks to more intuitively display the relationships between acupuncture methods, acupoints, and outcome measures; future research may consider these approaches to enhance data presentation. Additionally, outcomes were not stratified by acne severity, subtype, or patient characteristics, particularly in evaluating fire needle therapy’s specific advantages for moderate-to-severe cases. Furthermore, the absence of direct comparisons between different acupuncture modalities regarding their respective indications for acne subtypes somewhat limits the clinical applicability of findings. These aspects warrant further investigation to strengthen evidence-based recommendations.

## 6 Conclusion

This study provides a systematic synthesis of research characteristics, methodological quality, treatment protocols, outcome measures, and safety data regarding acupuncture for acne management. As a widely practiced complementary and alternative therapy globally, acupuncture offers acne patients a safe and effective non-pharmacological intervention, with various acupuncture modalities demonstrating considerable therapeutic potential. To enhance evidence quality, future research should prioritize multicenter, large-scale randomized controlled trials conducted in strict accordance with recognized reporting standards such as Consolidated Standards of Reporting Trials (CONSORT) (Moher et al., 2010). Future systematic reviews should focus on high-quality randomized controlled trials. A multidimensional efficacy evaluation system incorporating objective biological indicators should be established to minimize the bias associated with relying solely on subjective scales. Furthermore, future studies should integrate clinical quantitative measures, such as counts of inflammatory lesions and sebum secretion assessments, with biological sample analyses, including HPA-axis hormones, inflammatory mediators, and skin microcirculation imaging, to achieve a dynamic correlation between therapeutic outcomes and underlying mechanisms, thereby providing a scientific basis for clinical application. By optimizing study designs, refining outcome assessment methodologies, and deepening mechanistic investigations, these collective efforts will strengthen the evidence base for acupuncture therapy in dermatological practice and promote its international recognition as an evidence-based treatment option for acne vulgaris.

## Data Availability

The original contributions presented in the study are included in the article/Supplementary Material, further inquiries can be directed to the corresponding author.
