# Supplementary material for: Current state of research on acupuncture for acne: a scoping review
Source: Front Physiol. 2025 Oct 3;16:1661850. doi: 10.3389/fphys.2025.1661850 (PMC12532008; doi:10.3389/fphys.2025.1661850)
Supplement: Supplementary file 1 [file Table1.doc]

# Appendix 1: Search strategies for each database

| **Database** | **Search strategies** |
| --- | --- |
| PubMed | #1 Search: "Acne Vulgaris"[Mesh]  #2 Search: Acne[Title/Abstract]  #3 Search: ("Acne Vulgaris"[Mesh]) OR (Acne[Title/Abstract])  #4 Search: ("Acupuncture Therapy"[Mesh]) OR "Acupuncture"[Mesh]  #5 Search: (((((((((((Acupuncture needling[Title/Abstract]) OR (Electroacupuncture[Title/Abstract])) OR (Fire needling[Title/Abstract])) OR (Acupoint injection[Title/Abstract])) OR (Autohemotherapy[Title/Abstract])) OR (Acupoint embedding therapy[Title/Abstract])) OR (Auricular acupuncture[Title/Abstract])) OR (Auricular points[Title/Abstract])) OR (Bloodletting[Title/Abstract])) OR (Warm needling moxibustion[Title/Abstract])) OR (Moxibustion[Title/Abstract])) OR (Plum blossom needle therapy[Title/Abstract])  #6 Search: (("Acupuncture Therapy"[Mesh]) OR "Acupuncture"[Mesh]) OR ((((((((((((Acupuncture needling[Title/Abstract]) OR (Electroacupuncture[Title/Abstract])) OR (Fire needling[Title/Abstract])) OR (Acupoint injection[Title/Abstract])) OR (Autohemotherapy[Title/Abstract])) OR (Acupoint embedding therapy[Title/Abstract])) OR (Auricular acupuncture[Title/Abstract])) OR (Auricular points[Title/Abstract])) OR (Bloodletting[Title/Abstract])) OR (Warm needling moxibustion[Title/Abstract])) OR (Moxibustion[Title/Abstract])) OR (Plum blossom needle therapy[Title/Abstract]))  #7 Search: (("Acne Vulgaris"[Mesh]) OR (Acne[Title/Abstract])) AND ((("Acupuncture Therapy"[Mesh]) OR "Acupuncture"[Mesh]) OR ((((((((((((Acupuncture needling[Title/Abstract]) OR (Electroacupuncture[Title/Abstract])) OR (Fire needling[Title/Abstract])) OR (Acupoint injection[Title/Abstract])) OR (Autohemotherapy[Title/Abstract])) OR (Acupoint embedding therapy[Title/Abstract])) OR (Auricular acupuncture[Title/Abstract])) OR (Auricular points[Title/Abstract])) OR (Bloodletting[Title/Abstract])) OR (Warm needling moxibustion[Title/Abstract])) OR (Moxibustion[Title/Abstract])) OR (Plum blossom needle therapy[Title/Abstract])))  #8 Search: (("Acne Vulgaris"[Mesh]) OR (Acne[Title/Abstract])) AND ((("Acupuncture Therapy"[Mesh]) OR "Acupuncture"[Mesh]) OR ((((((((((((Acupuncture needling[Title/Abstract]) OR (Electroacupuncture[Title/Abstract])) OR (Fire needling[Title/Abstract])) OR (Acupoint injection[Title/Abstract])) OR (Autohemotherapy[Title/Abstract])) OR (Acupoint embedding therapy[Title/Abstract])) OR (Auricular acupuncture[Title/Abstract])) OR (Auricular points[Title/Abstract])) OR (Bloodletting[Title/Abstract])) OR (Warm needling moxibustion[Title/Abstract])) OR (Moxibustion[Title/Abstract])) OR (Plum blossom needle therapy[Title/Abstract]))) |
| EMBASE | #1 'acne vulgaris'/exp OR 'acne vulgaris'  #2 'acne':ab,ti  #3 #1 OR #2  #4 'acupuncture'/exp OR 'acupuncture'  #5 'acupuncture therapy':ab,ti OR 'acupuncture needling':ab,ti OR 'electroacupuncture':ab,ti OR 'fire needling':ab,ti OR 'auricular acupuncture':ab,ti OR 'acupoint injection':ab,ti OR 'autohemotherapy':ab,ti OR 'acupoint embedding therapy':ab,ti OR 'auricular points':ab,ti OR 'bloodletting':ab,ti OR 'warm needling moxibustion':ab,ti OR 'plum blossom needle therapy':ab,ti OR 'moxibustion':ab,ti  #6 #4 OR #5  #7 #3 AND #6 |
| Cochrane Library | #1 MeSH descriptor: [Acne Vulgaris] explode all trees  #2 (acne):ti,ab,kw  #3 #1 OR #2  #4 MeSH descriptor: [Acupuncture Therapy] explode all trees  #5 (Acupuncture):ti,ab,kw OR (Acupuncture needling):ti,ab,kw OR (Electroacupuncture):ti,ab,kw OR (Fire needling):ti,ab,kw OR (Auricular Acupuncture):ti,ab,kw OR (Acupoint injection):ti,ab,kw OR (Autohemotherapy):ti,ab,kw OR (Acupoint embedding therapy):ti,ab,kw OR (Auricular points):ti,ab,kw OR (Bloodletting):ti,ab,kw OR (Warm needling moxibustion):ti,ab,kw OR (Plum blossom needle therapy):ti,ab,kw OR (Moxibustion):ti,ab,kw  #6 #4 OR #5  #7 #3 AND #6 |
| Web of Science | #1 Acne Vulgaris (Topic)  #2 Acne Vulgaris OR Acne (Topic)  #3 Acne Vulgaris (Topic) or Acne (Topic)  #4 Acupuncture Therapy (Topic) or Acupuncture (Topic) or Acupuncture needling(Topic)or Electroacupuncture (Topic) or Fire needling (Topic) or AuricularAcupuncture (Topic) or Acupoint injection (Topic) or Autohemotherapy (Topic) orAcupoint embedding therapy (Topic) or Auricular points (Topic) or Bloodletting(Topic) or Warm needling moxibustion (Topic) or Plum blossom needle therapy or Moxibustion (Topic)  #5 #3AND #4 |
| AMED — by EBSCOhost | S1 SU Acne Vulgaris OR Su Acne  S2 SU acupuncture therapy OR SU ( acupuncture OR acupuncture needling OR electroacupuncture OR fire needling OR auricular acupuncture OR acupoint injection OR autohemotherapy OR acupoint embedding therapy OR auricular points OR bloodletting OR warm needling moxibustion OR plum blossom needle therapy OR moxibustion )  S3 (SU acupuncture therapy OR SU acupuncture OR acupuncture needling OR electroacupuncture OR fire needling OR auricular acupuncture OR acupoint injection OR autohemotherapy OR acupoint embedding therapy OR auricular points OR bloodletting OR warm needling moxibustion OR plum blossom needle therapy OR moxibustion) AND (S1 AND S2) |
| CNKI | （主题：寻常痤疮 + 痤疮 + 粉刺 + 青春痘）AND（主题：针灸 + 针刺 + 电针 + 火针 + 穴位注射 + 自血疗法 + 穴位埋线 + 耳针 + 耳穴 + 放血疗法 + 温针灸 + 艾灸 + 梅花针） |
| WanFang | (主题:(寻常痤疮 OR 痤疮 OR 粉刺 OR 青春痘) and 主题:(针灸 OR 针刺 OR 电针 OR 火针 OR 穴位注射 OR 自血疗法 OR 穴位埋线 OR 耳针 OR 耳穴 OR 放血疗法 OR 温针灸 OR 艾灸 OR 梅花针)) |
| Sinomed | #1 "寻常痤疮"[不加权:扩展]  #2 痤疮 OR 粉刺 OR 青春痘  #3 (#2) OR (#1)  #4 "针灸疗法"[不加权:扩展]  #5 针灸 OR 针刺 OR 电针 OR 火针 OR 穴位注射 OR 自血疗法 OR 穴位埋线 OR 耳针 OR 耳穴 OR 放血疗法 OR 温针灸 OR 艾灸 OR 梅花针  #6 (#5) OR (#4)  #7 ((#6) AND (#3)) AND 2014-2024[日期] |
| VIP | (U = 寻常痤疮 OR 痤疮 OR 粉刺 OR 青春痘 ) AND ( U = 针灸 OR 针刺 OR 电针 OR 火针 OR 穴位注射 OR 自血疗法 OR 穴位埋线 OR 耳针 OR 耳穴 OR 放血疗法 OR 温针灸 OR 艾灸 OR 梅花针) |
