# Supplementary material for: Current state of research on acupuncture for acne: a scoping review
Source: Front Physiol. 2025 Oct 3;16:1661850. doi: 10.3389/fphys.2025.1661850 (PMC12532008; doi:10.3389/fphys.2025.1661850)
Supplement: Supplementary file 2 [file Table2.docx]

**Appendix 2. Basic study characteristics**

| **IDX** | **Authors** | **Years** | **Country** | **region** | **Source** | **Journal rank** | **Research design** | **Funding** | **Language** |
| --- | --- | --- | --- | --- | --- | --- | --- | --- | --- |
|  | Mao Q R | 2024 | China | Fujian | Hunan Journal of Traditional Chinese Medicine | General publications | RCT | Yes | Chinese |
|  | Chen Z W | 2023 | China | Beijing | Journal of Sichuan of Traditional Chinese Medicine | General publications | RCT | No | Chinese |
|  | Fu Y H | 2023 | China | Guangdong | Lingnan Journal of Emergency Medicine | General publications | Before and after controlled trial | Yes | Chinese |
|  | Zhang M | 2023 | China | Tianjin | Journal of Practical Traditional Chinese Medicine | General publications | Case series | No | Chinese |
|  | Liang S | 2022 | China | Fujian | Heilongjiang Journal of Traditional Chinese Medicine | General publications | Case series | Yes | Chinese |
|  | Liu H | 2022 | China | Guangdong | Journal of Practical Traditional Chinese Medicine | General publications | Case report | No | Chinese |
|  | Dai P | 2022 | China | Jiangxi | Jiangxi Journal of Traditional Chinese Medicine | General publications | RCT | Yes | Chinese |
|  | Jiang X M | 2022 | China | Guangxi | Journal of External Therapy of Traditional Chinese Medicine | General publications | RCT | Yes | Chinese |
|  | Tan J Y | 2022 | China | Jilin | Chinese Medicine Modern Distance Education of China | General publications | NRCT | Yes | Chinese |
|  | Yang X D | 2022 | China | Hunan | Medicine | General publications | RCT | No | Chinese |
|  | Lao Y S | 2022 | China | Guangxi | Medicine | General publications | NRCT | No | Chinese |
|  | Jiao R M | 2022 | China | Beijing | Acupuncture in Medicine | SCI | RCT | No | English |
|  | Pang R K | 2021 | China | Guangxi | Chinese Acupuncture & Moxibustion | PKU、CSCD | RCT | Yes | Chinese |
|  | Yan X Z | 2021 | China | Shaanxi | Journal of Shaanxi University of Chinese Medicine | General publications | NRCT | Yes | Chinese |
|  | Wu F Q | 2021 | China | Shaanxi | Chinese Journal of Aesthetic Medicine | CSTPCD | Cohort study | No | Chinese |
|  | Zhao H Q | 2021 | China | Henan | Capital Medicine | General publications | RCT | No | Chinese |
|  | Zhang G P | 2021 | China | Hunan | Journal of Medical Aesthetice and Cosmetology | General publications | RCT | No | Chinese |
|  | Ji D D | 2021 | China | Shanxi | Kang Yi | General publications | Case series | No | Chinese |
|  | Jiang Z B | 2021 | China | Liaoning | Chinese Medicine Modern Distance Education of China | General publications | RCT | Yes | Chinese |
|  | Wang J R | 2021 | China | Shandong | World Journal of Acupuncture-Moxibustion | SCI | Case series | Yes | English |
|  | Jia J L | 2021 | China | Guangdong | Medicine | General publications | RCT | No | Chinese |
|  | Zhou L L | 2020 | China | Fujian | Journal of Shanxi University of Chinese Medicine | General publications | Before and after controlled trial | Yes | Chinese |
|  | He L X | 2020 | China | Guangdong | Journal of China Prescription Drug | General publications | RCT | No | Chinese |
|  | Chen L L | 2020 | China | Tianjin | Shaanxi Journal of Traditional Chinese Medicine | CSTPCD | SR | Yes | Chinese |
|  | Xiang Y L | 2020 | China | Sichuan | Chinese Journal of Aesthetic Medicine | CSTPCD | Cohort study | No | Chinese |
|  | Yan X Z | 2020 | China | Shaanxi | Hunan Journal of Traditional Chinese Medicine | General publications | Before and after controlled trial | Yes | Chinese |
|  | Yan X Z | 2020 | China | Shaanxi | Shaanxi Journal of Traditional Chinese Medicine | CSTPCD | NRCT | Yes | Chinese |
|  | Yan X Z | 2020 | China | Shaanxi | Clinical Research and Practice | General publications | NRCT | Yes | Chinese |
|  | Zhang P | 2020 | China | Beijing | Journal of Medical Aesthetice and Cosmetology | General publications | RCT | No | Chinese |
|  | Huang L K | 2019 | China | Liaoning | Guide of China Medicine | General publications | NRCT | No | Chinese |
|  | Jiang J F | 2019 | China | Henan | Traditional Chinese Medicinal Research | General publications | NRCT | No | Chinese |
|  | Li S S | 2019 | China | Sichuan | Electronic Journal of Clinical Medical Literature | General publications | Cohort study | No | Chinese |
|  | Fan P W | 2019 | China | Anhui | Traditional Chinese Medicine Rehabilitation | General publications | RCT | No | Chinese |
|  | Zhou L L | 2019 | China | Fujian | Journal of Gansu University of Chinese Medicine | General publications | Before and after controlled trial | Yes | Chinese |
|  | He G | 2019 | China | Liaoning | Journal of Aerospace Medicine | General publications | RCT | No | Chinese |
|  | Li M G | 2019 | China | Guangdong | Jilin Journal of Chinese Medicine | CSTPCD | NRCT | Yes | Chinese |
|  | Zuo Q Y | 2018 | China | Guizhou | Journal of Practical Traditional Chinese Internal Medicine | General publications | NRCT | Yes | Chinese |
|  | Gao Y L | 2018 | China | Gansu | Clinical Journal of Chinese Medicine | General publications | RCT | No | Chinese |
|  | Hu C X | 2018 | China | Guangdong | Acta Chinese Medicine | CSTPCD | RCT | Yes | Chinese |
|  | Geng H Y | 2018 | China | Henan | China Medical Cosmetology | General publications | RCT | No | Chinese |
|  | Lu W | 2018 | China | Jiangsu | Chinese Acupuncture & Moxibustion | PKU、CSCD | NRCT | Yes | Chinese |
|  | Mao J | 2018 | China | Hunan | Hunan Journal of Traditional Chinese Medicine | General publications | RCT | No | Chinese |
|  | Bai H H | 2018 | China | Yunnan | Yunnan Journal of Traditional Chinese Medicine and Materia Medica | General publications | Case series | No | Chinese |
|  | Huang M T | 2018 | China | Guangxi | Guangxi Journal of Traditional Chinese Medicine | General publications | RCT | No | Chinese |
|  | Du F X | 2018 | China | Gansu | Chinese Journal of Traditional Medical Science and Technology | General publications | RCT | No | Chinese |
|  | Gong X F | 2018 | China | Guangdong | Hunan Journal of Traditional Chinese Medicine | General publications | RCT | No | Chinese |
|  | Gao J | 2018 | China | Liaoning | Jilin Journal of Chinese Medicine | CSTPCD | RCT | Yes | Chinese |
|  | Sun Z B | 2018 | China | Guizhou | Medical Diet and Health | General publications | RCT | No | Chinese |
|  | Wang X Y | 2018 | China | Hubei | Health Guide | General publications | RCT | No | Chinese |
|  | Wang Y C | 2018 | China | Heilongjiang | Heilongjiang Journal of Traditional Chinese Medicine | General publications | Case series | No | Chinese |
|  | Cao L L | 2018 | China | Tianjin | Health Guide | General publications | RCT | No | Chinese |
|  | Lu J M | 2018 | China | Anhui | World Journal of Acupuncture - Moxibustion | SCI | RCT | No | English |
|  | Mansu S | 2018 | Australia |  | Advances in Integrative Medicine | SCI | SR | Yes | English |
|  | Li G F | 2017 | China | Guangdong | Clinical Journal of Chinese Medicine | General publications | RCT | No | Chinese |
|  | Liu D J | 2017 | China | Fujian | China Health Standard Management | General publications | RCT | No | Chinese |
|  | Huang J T | 2017 | China | Guangdong | China Journal of Traditional Chinese Medicine and Pharmacy | PKU、CSCD | RCT | Yes | Chinese |
|  | Yang G J | 2017 | China | Henan | China's Naturopathy | General publications | Case series | No | Chinese |
|  | Yang Y | 2017 | China | Jiangsu | Hunan Journal of Traditional Chinese Medicine | General publications | RCT | No | Chinese |
|  | Liu Y Y | 2017 | China | Guangdong | Heilongjiang Journal of Traditional Chinese Medicine | General publications | Case series | No | Chinese |
|  | Liao J G | 2017 | China | Henan | China Medical Cosmetology | General publications | NRCT | No | Chinese |
|  | Sun D | 2017 | China | Heilongjiang | Acta Chinese Medicine and Pharmacology | CSTPCD | RCT | Yes | Chinese |
|  | Zhang Y W | 2017 | China | Beijing | Hebei Journal of Traditional Chinese Medicine | CSTPCD | Before and after controlled trial | No | Chinese |
|  | Gao Y Y | 2017 | China | Sichuan | Journal of Frontiers of Medicine | General publications | RCT | No | Chinese |
|  | Chen Z Y | 2016 | China | Guizhou | Inner Mongolia Journal of Traditional Chinese Medicine | General publications | RCT | No | Chinese |
|  | Wu J Y | 2016 | China | Shanxi | Shanxi Medical Journal | General publications | Case series | No | Chinese |
|  | Qi M Y | 2016 | China | Fujian | Chinese Journal of Aesthetic Medicine | CSTPCD | RCT | No | Chinese |
|  | Lu J W | 2016 | China | Jiangsu | Heilongjiang Journal of Traditional Chinese Medicine | General publications | RCT | No | Chinese |
|  | Liu F | 2016 | China | Gansu | Journal of Practical Traditional Chinese Medicine | General publications | Case series | No | Chinese |
|  | Nong Q P | 2016 | China | Guangxi | Hunan Journal of Traditional Chinese Medicine | General publications | RCT | No | Chinese |
|  | Lin Z R | 2016 | China | Beijing | Western Journal of Traditional Chinese Medicine | CSTPCD | RCT | No | Chinese |
|  | Fan Y | 2016 | China | Henan | China Practical Medicine | General publications | RCT | No | Chinese |
|  | Liu Q Q | 2016 | China | Zhejiang | Yiyao Qianyan | General publications | RCT | No | Chinese |
|  | Fu Q F | 2016 | China | Shaanxi | World Journal of Acupuncture - Moxibustion | SCI | Case series | No | English |
|  | Zhang X M | 2015 | China | Jiangsu | Shanghai Journal of Acupuncture and Moxibustion | CSCD | RCT | No | Chinese |
|  | Sun H L | 2015 | China | Jilin | Journal of Changchun University of Chinese Medicine | CSTPCD | Case report | Yes | Chinese |
|  | Gao Y L | 2015 | China | Gansu | Journal of External Therapy of Traditional Chinese Medicine | General publications | Case series | No | Chinese |
|  | Wu J M | 2015 | China | Shanxi | Practical Clinical Journal of Integrated Traditional Chinese and Western Medicine | General publications | Case series | No | Chinese |
|  | Ai S Q | 2015 | China | Heilongjiang | World Latest Medicine Information | General publications | RCT | No | Chinese |
|  | Zuo Z | 2015 | China | Yunnan | Journal of Clinical Acupuncture and Moxibustion | CSTPCD | RCT | No | Chinese |
|  | Mo Q H | 2015 | China | Guangdong | Guiding Journal of Traditional Chinese Medicine and Pharmacy | CSTPCD | SR | No | Chinese |
|  | Yang S Y | 2015 | China | Fujian | Clinical Journal of Chinese Medicine | General publications | Before and after controlled trial | No | Chinese |
|  | Liu S M | 2015 | China | Shandong | Chinese Journal of Acupuncture and Moxibustion(Electronic Edition) | General publications | RCT | No | Chinese |
|  | Qin J L | 2015 | China | Shanxi | Clinical Journal of Chinese Medicine | General publications | Case series | No | Chinese |
|  | Yang Y C | 2015 | China | Gansu | Inner Mongolia Journal of Traditional Chinese Medicine | General publications | Case series | No | Chinese |
|  | Yang S Y | 2015 | China | Fujian | The World Clinical Medicine | General publications | RCT | No | Chinese |
|  | Yang D | 2015 | China | Shandong | Women's Health Research | General publications | Case series | No | Chinese |
|  | Zhang Y J | 2015 | China | Hebei | Traditional Chinese Medicine Rehabilitation | General publications | Case series | No | Chinese |
|  | Zhu J H | 2015 | China | Fujian | Journal of Practical Traditional Chinese Medicine | General publications | Case series | No | Chinese |
|  | Din J X | 2014 | China | Fujian | Journal of Yunnan University of Chinese Medicine | General publications | Before and after controlled trial | No | Chinese |
|  | Jiang Y M | 2014 | China | Tianjin | Inner Mongolia Journal of Traditional Chinese Medicine | General publications | Case series | No | Chinese |
|  | Li D | 2014 | China | Hubei | Inner Mongolia Journal of Traditional Chinese Medicine | General publications | Case report | No | Chinese |
|  | Sun Y | 2014 | China | Jilin | Chinese Medicine Modern Distance Education of China | General publications | NRCT | No | Chinese |
|  | Zhang L | 2014 | China | Beijing | Lishizhen Medicine and Materia Medica Research | PKU、CSCD | RCT | No | Chinese |
|  | Cheng X Y | 2014 | China | Jilin | China & Foreign Medical Treatment | General publications | Cohort study | No | Chinese |
|  | Dong L J | 2014 | China | Hebei | Traditional Chinese Medicine | General publications | Case series | No | Chinese |
|  | Wang G A | 2014 | China | Henan | Acta Chinese Medicine | CSTPCD | RCT | No | Chinese |
|  | Li G Q | 2014 | China | Shandong | Journal of Sichuan of Traditional Chinese Medicine | General publications | Case series | Yes | Chinese |
|  | Cui X X | 2014 | China | Shandong | Hunan Journal of Traditional Chinese Medicine | General publications | Case series | No | Chinese |
|  | You M L | 2014 | China | Guangdong | Shanghai Journal of Acupuncture and Moxibustion | CSCD | NRCT | No | Chinese |
|  | Pang K Y | 2014 | China | Henan | China's Naturopathy | General publications | Case series | No | Chinese |
|  | Qin X Y | 2014 | China | Henan | China's Naturopathy | General publications | NRCT | No | Chinese |
|  | Qu X M | 2014 | China | Jilin | China Medical Cosmetology | General publications | Case series | No | Chinese |
|  | Wu J | 2014 | China | Sichuan | Inner Mongolia Journal of Traditional Chinese Medicine | General publications | NRCT | No | Chinese |
|  | Jiang M | 2014 | China | Hubei | Chinese Acupuncture & Moxibustion | PKU、CSCD | RCT | No | Chinese |
|  | Zhang X P | 2014 | China | Guangdong | Shanghai Journal of Acupuncture and Moxibustion | CSCD | Before and after controlled trial | No | Chinese |
|  | Liu N Y | 2014 | China | Hubei | Hubei Journal of Traditional Chinese Medicine | General publications | Case series | No | Chinese |
|  | Huang Y S | 2014 | China | Hubei | Hubei Journal of Traditional Chinese Medicine | General publications | Case series | No | Chinese |
|  | Xu J R | 2014 | China | Hubei | Modern Hospital | General publications | Case series | No | Chinese |
|  | Jiao R | 2014 | China | Liaoning | Journal of Liaoning University of Traditional Chinese Medicine | CSTPCD | Case series | Yes | Chinese |
|  | Gu C Y | 2014 | China | Jiangsu | Shanghai Journal of Acupuncture and Moxibustion | CSCD | NRCT | No | Chinese |
|  | Zeng X | 2014 | China | Guizhou | Clinical Journal of Chinese Medicine | General publications | Case series | No | Chinese |
|  | Tao X F | 2014 | China | Jiangsu | Jiangsu Journal of Traditional Chinese Medicine | CSTPCD | NRCT | No | Chinese |
|  | Qiu B W | 2014 | China | Gansu | Traditional Chinese Medicine Rehabilitation | General publications | Case series | No | Chinese |
|  | Guan X L | 2014 | China | Henan | China's Naturopathy | General publications | Case series | No | Chinese |

1. Mao Q, Jing Y, Tong B, et al. Acupuncture combined with moxa stick clear-through point moxibustion for the treatment of 30 cases of common acne vulgaris. Hunan Journal of Traditional Chinese Medicine, 2024;40(7):78-81.

2. Chen Z, Du L. Exploring the clinical value of acupuncture in treating common acne based on color models. Sichuan Traditional Chinese Medicine, 2023;41(11):190-193.

3. Fu Y. Analysis of "treating from the heart" with heart acupoint pricking and cupping combined with micro-fire needle intervention for acne in young people. Lingnan Emergency Medicine Journal, 2023;28(6):634-636.

4. Zhang M. Treatment of 32 cases of acne with bloodletting and cupping. Journal of Practical Traditional Chinese Medicine, 2023;39(5):988.

5. Liu H, Liu J. Local bloodletting therapy combined with thermal moxibustion for the treatment of moderate to severe acne: a case report. Journal of Practical Traditional Chinese Medicine, 2022;38(12):2210-2211.

6. Dai P, Hu F, Ding S, et al. Clinical observation of thermal moxibustion combined with fire needle for the treatment of moderate acne with yang deficiency. Jiangxi Traditional Chinese Medicine, 2022;53(2):57-59.

7. Jiang X, Xie W, Huang S, et al. Treatment of 30 cases of common acne with Zhu Lian acupuncture combined with deep needling of Zusanli and Fenglong points. Journal of Traditional Chinese Medicine External Therapy, 2022;31(2):89-91.

8. Tan J, Peng M, Zhou Y, et al. Clinical observation of facial needling combined with cupping and pricking of back shu points for the treatment of common acne. Modern Distance Education of Traditional Chinese Medicine, 2022;20(2):122-124.

9. Yang X, Liang S, Chen K, et al. Clinical experience with facial needling combined with auricular acupressure and distant acupoint embedding for the treatment of adolescent acne. Chinese Science and Technology Journal Database (Abstract Edition) Medicine and Health, 2022;(5):53-55.

10. Lao Y. Clinical analysis of auricular acupressure combined with fire needle for the treatment of acne. Chinese Science and Technology Journal Database (Full-text Edition) Medicine and Health, 2022;(2):232-235.

11. Liang S. Clinical efficacy observation of self-designed five viscera unblocking acupuncture for the treatment of 85 cases of acne. Heilongjiang Traditional Chinese Medicine, 2022;51(5):106-108.

12. Jiao R, Zhai X, Zhang X, et al. Efficacy of acupuncture in improving symptoms and quality of life of patients with acne vulgaris: a randomized sham acupuncture-controlled trial. Acupuncture in Medicine, 2022;40(5):453-462.

13. Peng R, Fan Y, He C, et al. Efficacy and mechanism of Zhu Lian inhibitory type I acupuncture for the treatment of acne with spleen and stomach damp-heat syndrome. Chinese Acupuncture & Moxibustion, 2021;41(11):1236-1240.

14. Yan X, Zheng F, Huang L, et al. Clinical study on autologous blood therapy for moderate to severe common acne. Journal of Shaanxi University of Traditional Chinese Medicine, 2021;44(6):97-100.

15. Wu F, Wang L, Liu M, et al. Comparative observation of efficacy and safety between clearing therapy and fire needle for the treatment of common acne. Chinese Aesthetic Medicine, 2021;30(5):115-118.

16. Zhao H, Yu X. Effects of warm acupuncture combined with bloodletting therapy on skin lesion scores, quality of life, and skin barrier function in patients with acne. Capital Food and Medicine, 2021;28(13):140-141.

17. Zhang G, Liu R. Clinical efficacy of autologous blood therapy combined with pricking and cupping for the treatment of acne. Medical Aesthetics and Cosmetology, 2021;30(3):59.

18. Ji D, Li L, Fu X. Clinical observation of autologous blood therapy combined with pricking and cupping for the treatment of 60 cases of acne. Kangyi, 2021;(18):171-172.

19. Jiang Z, Ma Y, Zhao S, et al. Clinical observation of traditional Chinese medicine acupoint plaster for the treatment of mild acne. Modern Distance Education of Traditional Chinese Medicine, 2021;19(21):114-117.

20. Jia J, Lu D. Observing the clinical efficacy of fire needle therapy for acne. Chinese Science and Technology Journal Database (Full-text Edition) Medicine and Health, 2021;(7):48-49.

21. Wang J, Sun X, Zhang X, et al. Twenty-eight cases of facial acne treated by quick puncture with collateral-pricking cupping. World Journal of Acupuncture - Moxibustion, 2021;31(4):262-265.

22. Zhou L, Pan X, Sa Z, et al. Effects of electroacupuncture at Hegu point on skin temperature along the forearm meridian of the large intestine in patients with acne. Journal of Shanxi University of Traditional Chinese Medicine, 2020;21(4):276-278.

23. He L. Efficacy study of regulating spirit and tonifying spleen acupuncture for patients with common facial acne. Chinese Prescription Medicine, 2020;18(5):143-144.

24. Chen L, Du Y, Li J, et al. Systematic review of the efficacy comparison between acupuncture and Western medicine for the treatment of acne. Shaanxi Traditional Chinese Medicine, 2020;41(5):682-685.

25. Xiang Y, Xu T. Observation of the efficacy of fire needle combined with acupoint embedding for the treatment of cystic acne and its effect on skin barrier function. Chinese Aesthetic Medicine, 2020;29(4):138-141.

26. Yan X, Qu Q, Bi Y, et al. Clinical observation of autologous blood acupoint injection therapy for the treatment of 28 cases of common facial acne. Hunan Journal of Traditional Chinese Medicine, 2020;36(3):74-75.

27. Yan X, Qu Q, Huang L, et al. Clinical efficacy of autologous blood therapy for acne and its effect on serum inflammatory factors. Shaanxi Traditional Chinese Medicine, 2020;41(1):108-110.

28. Yan X, Qu Q, Huang L, et al. Effects of autologous blood therapy on common acne and its influence on immune function. Clinical Medical Research and Practice, 2020;5(1):129-131.

29. Zhang P. Clinical effects of acupuncture for acne and its improvement on clinical symptoms. Medical Aesthetics and Cosmetology, 2020;29(21):127.

30. Huang L. Clinical observation of autologous blood therapy combined with pricking and cupping for the treatment of 49 cases of common acne. China Medical Guide, 2019;17(30):223-224.

31. Jiang J. Fire needle therapy for 60 cases of facial pustular acne. Traditional Chinese Medicine Research, 2019;32(7):55-57.

32. Li S. Observing the effects of fire needle therapy for acne. Clinical Medicine and Pharmacy Journal, 2019;6(52):45-46.

33. Fan P, Li B. Clinical efficacy observation of acupuncture combined with local surrounding needling for adolescent acne. Massage and Rehabilitation Medicine, 2019;10(14):27-29.

34. Zhou L, Sa Z, Zhu X, et al. Effects of electroacupuncture at Hegu and Quchi points on facial microcirculatory blood flow in patients with acne. Journal of Gansu University of Traditional Chinese Medicine, 2019;36(2):8-11.

35. He G, Zhang Q. Observation of the efficacy of fire needle combined with cupping for the treatment of cystic acne. Aerospace Medicine Journal, 2019;30(2):212-213.

36. Li M, Tang C. Treatment of acne with surrounding needling at Ashi points combined with embedding at back shu points. Jilin Traditional Chinese Medicine, 2019;(8):1098-1101.

37. Zuo Q. Randomized parallel controlled study of bloodletting therapy for female acne (lung-stomach damp-heat syndrome). Practical Journal of Traditional Chinese Medicine Internal Medicine, 2018;32(12):47-51.

38. Gao Y, An D, Li X. Clinical study on comprehensive diagnosis and treatment of common adolescent acne with needling at San Ying points as the main method. Traditional Chinese Medicine Clinical Research, 2018;10(33):69-72.

39. Hu C, Li S, Chen X, et al. Clinical study on fire needle combined with electroacupuncture for the treatment of lung meridian wind-heat type acne. Journal of Traditional Chinese Medicine, 2018;33(9):1819-1822.

40. Geng H, Liu L, Han Y. Clinical experience with acupuncture in improving the treatment effects of facial acne. Chinese Medical Cosmetology, 2018;8(8):71-73.

41. Lu W, Zhu L, Tian T, et al. Comprehensive treatment of post-adolescent acne in women with acupoint embedding, fire needle, and auricular needle, and its effect on serum hormone levels. Chinese Acupuncture & Moxibustion, 2018;38(8):833-838.

42. Mao J, Chen L, Wang J, et al. Observation of the efficacy of autologous blood therapy combined with pricking and cupping for the treatment of 51 cases of acne. Hunan Journal of Traditional Chinese Medicine, 2018;34(6):75-76.

43. Bai H, Wang Y, Tong H. Nursing observation of acupoint autologous blood therapy combined with pricking and cupping for the treatment of stubborn comedones. Yunnan Journal of Traditional Chinese Medicine, 2018;39(6):89-90.

44. Huang M, Zheng G, Wei B, et al. Observation of the efficacy of plum blossom needle bloodletting for second-degree common acne. Guangxi Traditional Chinese Medicine, 2018;41(3):38-39.

45. Du F, Zhang X, Du X. Clinical efficacy observation of cooling and purging acupuncture for acne. Chinese Traditional Chinese Medicine Science and Technology, 2018;25(2):276-278.

46. Gong X, Zhang L. Clinical observation of fire needle combined with back shu points pricking and bloodletting for the treatment of 30 cases of acne. Hunan Journal of Traditional Chinese Medicine, 2018;34(1):89-91.

47. Gao J. Fire needle combined with pricking and cupping for the treatment of pustular acne. Jilin Traditional Chinese Medicine, 2018;38(1):109-112.

48. Sun Z, Chen Z, Shu K, et al. Observation and study of the application effects of fire needle therapy for acne. Medical Diet Therapy and Health, 2018;(7):159, 162.

49. Wang X. Therapeutic effects of fire needle therapy for acne. Health and Wellness Guide, 2018;(50):223.

50. Wang Y. Clinical observation of pricking and cupping for the treatment of lung meridian heat-retention type acne (with 113 cases). Heilongjiang Traditional Chinese Medicine, 2018;48(3):89-90.

51. Cao L, Wang B, Liu C, et al. Application effects of auricular points combined with scraping in traditional Chinese medicine cosmetology. Health and Wellness Guide, 2018;(11):333-334.

52. Mansu S S Y, Liang H, Parker S, et al. Acupuncture for Acne Vulgaris: A Systematic Review and Meta-Analysis. Evidence-based Complementary and Alternative Medicine, 2018;2018.

53. Lu J, Lu Z. Acupuncture combined with cupping and circling moxibustion for 40 cases of acne. World Journal of Acupuncture - Moxibustion, 2018;28(2):134-136.

54. Gao Y. Clinical study on the treatment of confluent acne with facial pricking and flash cupping combined with fire needle. Medical Frontier, 2017;7(35):301-302.

55. Li G. Exploring the clinical effects of fire needle therapy for nodulocystic acne. Traditional Chinese Medicine Clinical Research, 2017;9(35):40-41.

56. Liu D, Tian X, Wu J. Observation of the efficacy of acupuncture combined with Shenque salt moxibustion for the treatment of acne with Chong Ren disorder. China Health Standard Management, 2017;8(24):112-113.

57. Huang J, Luo X, Tang C. A randomized controlled trial of acupoint embedding combined with conventional acupuncture for the treatment of acne. Chinese Journal of Traditional Chinese Medicine, 2017;32(8):3825-3828.

58. Yang G. Treatment of 48 cases of lung-stomach heat-retention type acne with acupoint embedding using the Bu Mu Xia Zi method. Chinese Folk Therapy, 2017;25(9):22-23.

59. Yang Y, Wang M. Observation of the efficacy of acupoint embedding combined with fire needle for the treatment of 50 cases of common acne. Hunan Journal of Traditional Chinese Medicine, 2017;33(4):83-84.

60. Liu Y, Wang X. Treatment of 37 cases of facial acne in women with acupoint injection combined with timed bloodletting and cupping. Heilongjiang Traditional Chinese Medicine, 2017;46(1):61.

61. Liao J, Li J. Observing the efficacy of sharp hook needle drainage and bloodletting for the repair of skin lesions in nodular acne. Chinese Medical Cosmetology, 2017;7(10):69-71.

62. Sun D, Sun Q, Sun W, et al. Observation of the effects of body acupuncture combined with plum blossom needle therapy for the treatment of acne with Chong Ren disorder. Journal of Traditional Chinese Medicine, 2017;45(3):51-53.

63. Zhang Y, Hua H, Tao S. Observation of the efficacy of traditional Chinese medicine external application at Yongquan point for the treatment of common acne. Hebei Traditional Chinese Medicine, 2017;39(2):234-237.

64. Chen Z, Yang F. Analysis of the effects of acupuncture combined with bloodletting and cupping for the treatment of common acne. Inner Mongolia Traditional Chinese Medicine, 2016;35(13):133.

65. Wu J. Clinical observation of bloodletting combined with fine needle therapy for facial acne. Shanxi Medical Journal, 2016;45(20):2434-2435.

66. Qi M, Lin B, Xie Z, et al. Clinical observation of 34 cases of cystic acne in adolescents treated with fire needle cauterization. Chinese Aesthetic Medicine, 2016;25(6):92-94.

67. Lu J. Observation of the efficacy of autologous blood therapy for common acne. Heilongjiang Traditional Chinese Medicine, 2016;45(3):34-35.

68. Liu F. Clinical observation of fire needle combined with cupping for the treatment of cystic and nodular acne. Practical Journal of Traditional Chinese Medicine, 2016;32(4):365-366.

69. Nong Q, Tang S, Ruan J, et al. Clinical observation of 30 cases of acne treated with acupoint embedding combined with back cupping. Hunan Journal of Traditional Chinese Medicine, 2016;32(3):106-107.

70. Lin Z, Zhou W, You S, et al. Clinical observation of bloodletting combined with acupoint injection for the treatment of acne. Western Journal of Traditional Chinese Medicine, 2016;29(3):102-104.

71. Fan Y. Evaluation of the clinical efficacy of fire needle therapy for acne. Chinese Practical Medicine, 2016;11(3):275-276.

72. Liu Q, Huang W, Wang W, et al. Fire needle therapy for facial acne. Medical Frontier, 2016;6(18):179-180.

73. Fu Q, Zhang Q, Duan B, et al. Prick-bloodletting combined with cupping on the back for 52 cases of acne. World Journal of Acupuncture - Moxibustion, 2016;26(4):61-64.

74. Zhang X. Clinical observation of autologous blood therapy combined with pricking and cupping for the treatment of common acne. Shanghai Journal of Acupuncture and Moxibustion, 2015;34(12):1208-1210.

75. Sun H, Xiong G. Treatment of adolescent common acne with facial needling combined with back shu points pricking and cupping. Journal of Changchun University of Traditional Chinese Medicine, 2015;31(6):1171-1172.

76. Gao Y, An D, Li X. Acupuncture at San Ying points as the main method for the comprehensive diagnosis and treatment of 56 cases of common adolescent acne. Journal of Traditional Chinese Medicine External Therapy, 2015;24(6):11.

77. Wu J. Observation of the efficacy of autologous blood acupoint injection combined with bloodletting for the treatment of 37 cases of acne (heat-toxin type). Practical Clinical Journal of Integrated Traditional Chinese and Western Medicine, 2015;15(9):79-80.

78. Ai S. Observation of the efficacy of back shu points pricking and cupping combined with moxibustion for the treatment of patients with intestinal and stomach damp-heat type acne. World Latest Medical Information Abstracts, 2015;15(76):78.

79. Zuo Z, Guan Z. Observation of the efficacy of autologous blood acupoint injection combined with bloodletting for the treatment of acne. Journal of Clinical Acupuncture and Moxibustion, 2015;31(7):42-44.

80. Mo Q, Liang L, Liao J, et al. Meta-analysis of randomized controlled clinical studies on acupuncture for acne. Traditional Chinese Medicine Guide, 2015;21(12):76-83.

81. Yang S, Xu J, Pan X, et al. Observation of the effect of single needling at Hegu point on infrared thermal imaging of the face and mouth in patients with common acne. Traditional Chinese Medicine Clinical Research, 2015;7(15):108-110.

82. Liu S, Shi B. Efficacy of acupuncture for acne and its impact on immune function. Chinese Journal of Acupuncture and Moxibustion, 2015;4(2):1-3.

83. Qin J, Zhang W. Autologous blood therapy combined with body acupuncture for 35 cases of acne. Traditional Chinese Medicine Clinical Research, 2015;7(7):58-59.

84. Yang Y. Clinical observation of 99 cases of acne treated with acupuncture. Inner Mongolia Traditional Chinese Medicine, 2015;34(1):72.

85. Yang S. Clinical observation of 35 cases of common acne treated with acupoint embedding combined with bloodletting. World Clinical Medicine, 2015;9(11):262-263.

86. Yang D, Tan W. Clinical observation of abdominal acupuncture combined with hand acupuncture and bloodletting for the treatment of acne. Journal of International and Chinese Women's Health, 2015;(6):71-72.

87. Zhang Y, Yang Z. Observation of the efficacy of scraping combined with balancing acupuncture for the treatment of acne. Massage and Rehabilitation Medicine, 2015;6(9):40-41.

88. Zhu J, Shen G, Ma X. Treatment of 63 cases of acne with acupoint bloodletting. Practical Journal of Traditional Chinese Medicine, 2015;31(2):138.

89. Ding J, Lin X, Chen Y. Observation of the efficacy of acupuncture at "opening four gates" for the treatment of lung meridian wind-heat type common acne. Journal of Yunnan University of Traditional Chinese Medicine, 2014;37(6):30-31.

90. Jiang Y. Treatment of 50 cases of adolescent acne with pricking and cupping combined with auricular therapy. Inner Mongolia Traditional Chinese Medicine, 2014;33(34):70.

91. Li D. Two cases of acne treated with auricular bloodletting therapy in traditional Chinese medicine. Inner Mongolia Traditional Chinese Medicine, 2014;33(34):91.

92. Qu X, Chu Y. Exploring the application effects of auricular points combined with scraping in traditional Chinese medicine cosmetology. Chinese Medical Cosmetology, 2014;4(4):172-174.

93. Sun Y. Treatment of 32 cases of comedones with auricular tip combined with back shu points needling and bloodletting for lung meridian wind-heat syndrome. Modern Distance Education of Traditional Chinese Medicine, 2014;12(23):69-70.

94. Zhang L, Wu F, Xue L, et al. Comparative study of electric plum blossom needle tapping therapy for common acne. Shizhen Guo Yi Guo Yao, 2014;25(11):2817-2818.

95. Cheng X. Efficacy study of acupuncture for adolescent acne. Chinese and Foreign Medical Treatment, 2014;33(32):20-21.

96. Dong L. Observation of the efficacy of auricular endocrine points and auricular tip needling and bloodletting for the treatment of 45 cases of acne. Massage and Rehabilitation Medicine, 2014;5(11):125-126.

97. Wang G, Chen S. Clinical study on acupoint embedding for the treatment of acne. Journal of Traditional Chinese Medicine, 2014;29(11):1695-1696.

98. Li G, Zhang W, Yang D. Observation of the efficacy of auricular acupressure combined with Feishu and Dazhui pricking and cupping for the treatment of 38 cases of common acne. Sichuan Traditional Chinese Medicine, 2014;32(10):152-153.

99. Cui X, Ning K, Liu J, et al. Clinical observation of 39 cases of acne treated with Dazhui pricking and cupping combined with auricular acupressure. Hunan Journal of Traditional Chinese Medicine, 2014;30(9):94-95.

100. You M, Liu G. Observation of the efficacy of tonifying and unblocking acupuncture for facial acne. Shanghai Journal of Acupuncture and Moxibustion, 2014;33(9):836-837.

101. Pang K, Qin X, Hou Q, et al. Clinical observation of 60 cases of blood stasis and phlegm coagulation type acne treated with acupoint embedding. Chinese Folk Therapy, 2014;22(9):14-15.

102. Qin X, Pang K, Hou Q. Autologous blood therapy for 35 cases of comedones. Chinese Folk Therapy, 2014;22(8):22.

103. Wu J, Liu T, Wu J. Clinical observation of the efficacy of acupoint embedding combined with bloodletting for the treatment of lung meridian wind-heat type acne. Inner Mongolia Traditional Chinese Medicine, 2014;33(22):55.

104. Jiang M, Zeng X, Wang W. Observation of the efficacy of fire needle therapy for moderate to severe common acne. Chinese Acupuncture & Moxibustion, 2014;34(7):663-666.

105. Zhang X, Xue D, Tong A, et al. Observation of the efficacy of acupuncture for regulating yang deficiency constitution in the treatment of upper heat and lower cold type acne. Shanghai Journal of Acupuncture and Moxibustion, 2014;33(7):648-651.

106. Liu N. Treatment of acne with nasal needling combined with bloodletting. Hubei Journal of Traditional Chinese Medicine, 2014;36(7):59.

107. Huang Y, Chen M. Treatment of 60 cases of common acne with triple needle therapy. Hubei Journal of Traditional Chinese Medicine, 2014;36(7):62-63.

108. Xu J. Clinical observation of 35 cases of acne treated with scraping combined with electroacupuncture. Modern Hospital, 2014;14(5):63-64.

109. Jiao J, Zhang Y, Zhang X, et al. Analysis of the application of auricular points combined with scraping in traditional Chinese medicine cosmetology. Liaoning University of Traditional Chinese Medicine Journal, 2014;16(4):98-100.

110. Gu C, Huang J, Che Z. Clinical observation of the efficacy of comprehensive acupuncture with main focus on poking needle for acne. Shanghai Journal of Acupuncture and Moxibustion, 2014;33(2):132-134.

111. Zeng X, Yang D, Xu L, et al. Clinical observation of the efficacy of scraping combined with autologous blood therapy for acne. Traditional Chinese Medicine Clinical Research, 2014;6(1):52-54.

112. Tao X, Wang L. Clinical observation of 33 cases of spleen and stomach damp-heat type acne treated with acupuncture and cupping. Jiangsu Traditional Chinese Medicine, 2014;46(1):61-62.

113. Qiu B, Su H, Zhang Z, et al. Autologous blood acupoint injection combined with bloodletting for the treatment of 46 cases of facial acne. Massage and Rehabilitation Medicine, 2014;5(1):199.

114. Guan X. Treatment of 30 cases of acne with facial composite therapy combined with body acupuncture. Chinese Folk Therapy, 2014;22(4):42.
