# Supplementary material for: Current state of research on acupuncture for acne: a scoping review
Source: Front Physiol. 2025 Oct 3;16:1661850. doi: 10.3389/fphys.2025.1661850 (PMC12532008; doi:10.3389/fphys.2025.1661850)
Supplement: Supplementary file 4 [file Table3.docx]

**Appendix 3**

**Characteristics of included studies**

| **Study characteristics** | **Author and year** | **Disease** | **Sample size** | | **Interventions** | | **Randomization** | **Blinding** | **Evidence grade** |
| --- | --- | --- | --- | --- | --- | --- | --- | --- | --- |
|  |  |  | **T** | **C** | **T** | **C** |  |  |  |
| **RCT** | Zhang L（2014） | Acne | 20 | 20 | Electric plum blossom needle | Western medicine (topical) | Random number table | / | **IIb** |
|  | Wang G A（2014） | Acne | 66 | 66 | Acupoint catgut embedding | Western medicine (oral + topical) | Random number table | / |  |
|  | Jiang M（2014） | Acne | 30 | 30 | Fire needling therapy | Western medicine (oral + topical) | Random number table | / |  |
|  | Zhang X M（2015） | Acne | 28 | 28 | Pricking-cupping therapy+Autologous blood therapy | Western medicine (oral + topical) | Random number table | / |  |
|  | Ai S Q（2015） | Acne - Dampness-Heat Pattern of the Spleen and Stomach | 30 | 30 | Pricking-cupping therapy+moxibustion | Western medicine (topical) | Randomization method not specified | / |  |
|  | Zuo Z（2015） | Acne | 30 | 30 | Pricking-cupping therapy+Autologous blood therapy | Western medicine (oral) | Random number table | / |  |
|  | Liu S M（2015） | Acne | 60 | 58 | Filiform needling therapy | Western medicine (oral) | Random number table | / |  |
|  | Yang S Y（2015） | Acne | 35 | 35 | Pricking-cupping therapy+Acupoint catgut embedding | Western medicine (oral) | Random number table | / |  |
|  | Chen Z Y（2016） | Acne | 30 | 30 | Filiform needling therapy+Pricking-cupping therapy | Western medicine (oral) | Randomization method not specified | / |  |
|  | Qi M Y（2016） | Acne - Adolescent Type | 34 | 32 | Fire needling therapy | acne extractor | Randomization method not specified | / |  |
|  | Lu J W（2016） | Acne | 34 | 34 | Autologous blood therapy | Western medicine (oral) | Randomization method not specified | / |  |
|  | Nong Q P（2016） | Acne | 30 | 30 | Acupoint catgut embedding+Cupping therapy | Western medicine (oral + topical) | Randomization method not specified | / |  |
|  | Lin Z R（2016） | Acne | 56 | 56 | Bloodletting therapy+Pricking-cupping therapy+Acupoint injection | Western medicine (oral + topical) | Random number table | / |  |
|  | Fan Y（2016） | Acne | 25 | 26 | Fire needling therapy | Western medicine (topical) | lottery method | / |  |
|  | Liu Q Q（2016） | Acne | 30 | 30 | Fire needling therapy | Western medicine (topical) | Randomization method not specified | / |  |
|  | Li G F（2017） | Acne | 50 | 50 | Fire needling therapy+moxibustion | Western medicine (topical) | Randomization method not specified | / |  |
|  | Liu D J（2017） | acne - Chong Ren disharmony pattern | 30 | 30 | Filiform needling therapy+moxibustion | Western medicine (oral) | computer-generated randomization | / |  |
|  | Huang J T（2017） | Acne | 48 | 47 | Filiform needling therapy+Acupoint catgut embedding | Western medicine (oral) | Random number table | / |  |
|  | Yang Y（2017） | Acne | 50 | 50 | Acupoint catgut embedding+Fire needling therapy | Western medicine (oral + topical) | Randomization method not specified | / |  |
|  | Sun D（2017） | acne - Chong Ren disharmony pattern | 40 | 40 | Filiform needling therapy | Western medicine (oral) | Randomization method not specified | / |  |
|  | Gao Y Y（2017） | Acne | 25 | 25 | Pricking-cupping therapy+Fire needling therapy | Western medicine (oral) | Random number table | / |  |
|  | Gao Y L（2018） | Acne | 38 | 37 | Filiform needling therapy+Cupping therapy | Western medicine (topical) | lottery method | / |  |
|  | Hu C X（2018） | Acne - Lung Meridian Wind-Heat Pattern | 31 | 30 | Electroacupuncture therapy+Fire needling therapy | Chinese medicine (oral) | Random number table | / |  |
|  | Geng H Y（2018） | Acne | 25 | 25 | Filiform needling therapy | Western medicine (topical) | Random number table | / |  |
|  | Mao J（2018） | Acne | 51 | 51 | Autologous blood therapy+Pricking-cupping therapy | Western medicine (oral + topical) | Random number table | / |  |
|  | Huang M T（2018） | Acne | 16 | 20 | plum blossom needle | Western medicine (topical) | Randomization method not specified | / |  |
|  | Du F X（2018） | Acne | 30 | 30 | Filiform needling therapy | Western medicine (topical) | Random number table | / |  |
|  | Gong X F（2018） | Acne | 31 | 31 | Fire needling therapy+Pricking-cupping therapy | Western medicine (oral) | Randomization method not specified | / |  |
|  | Gao J（2018） | Acne | 36 | 36 | Fire needling therapy+Pricking-cupping therapy | Western medicine (oral) | Random number table | / |  |
|  | Sun Z B（2018） | Acne | 51 | 51 | Fire needling therapy | Western medicine (topical) | computer-generated randomization | / |  |
|  | Wang X Y（2018） | Acne | 24 | 27 | Fire needling therapy | Western medicine (topical) | lottery method | / |  |
|  | Cao L L（2018） | Acne | 60 | 60 | Bloodletting therapy+Gua sha | Chinese patent medicine(oral) | lottery method | / |  |
|  | Lu J M（2018） | Acne | 40 | 30 | Filiform needling therapy+Cupping therapy+moxibustion | Western medicine (oral) | Randomization method not specified | / |  |
|  | Fan P W（2019） | Acne | 30 | 30 | Filiform needling therapy | Western medicine (topical) | Random number table | / |  |
|  | He G（2019） | Acne | 35 | 35 | Fire needling therapy+Cupping therapy | Western medicine (oral) | Random number table | / |  |
|  | He L X（2020） | Acne | 50 | 50 | Filiform needling therapy | Western medicine (oral) | Randomization method not specified | / |  |
|  | Zhang P（2020） | Acne | 30 | 30 | Filiform needling therapy+auricular acupressure | Chinese medicine (oral) | lottery method | / |  |
|  | Pang R K（2021） | Acne - Spleen-Stomach Dampness-Heat Pattern | 41 | 41 | Filiform needling therapy | Western medicine (oral + topical) | Random number table | single-blind |  |
|  | Zhao H Q（2021） | Acne | 48 | 48 | Pricking-cupping therapy+warm needle acupuncture | Western medicine (oral) | Random number table | / |  |
|  | Zhang G P（2021） | Acne | 50 | 50 | Pricking-cupping therapy+Autologous blood therapy | Western medicine (oral + topical) | Random number table | / |  |
|  | Jiang Z B（2021） | Acne - Lung Meridian Wind-Heat Pattern | 15 | 15 | Acupoint application | blank patch | Random number table | / |  |
|  | Jia J L（2021） |  | 40 | 40 | Fire needling therapy | Western medicine (oral + topical) | computer-generated random sampling | / |  |
|  | Dai P（2022） | cne - Yang deficiency pattern | 60 | 60 | moxibustion+Fire needling therapy | Western medicine (oral + topical) | computer-generated randomization | / |  |
|  | Jiang X M（2022） | Acne | 30 | 30 | Filiform needling therapy | Western medicine (topical) | Random number table | / |  |
|  | Yang X D（2022） | Acne | 25 | 25 | Filiform needling therapy+auricular acupressure+Acupoint catgut embedding | Western medicine (topical) | Random number table | / |  |
|  | Jiao R M（2022） | Acne | 50 | 50 | Filiform needling therapy | sham acupuncture | computer-generated randomization | double-blind |  |
|  | Chen Z W（2023） | Acne | 40 | 40 | Filiform needling therapy | Western medicine (oral) | Random number table | / |  |
|  | Mao Q R（2024） | Acne | 30 | 30 | Filiform needling therapy+moxibustion | Western medicine (topical) | Random number table | / |  |
| **NRCT** | Sun Y（2014） | Acne - Lung Meridian Wind-Heat Pattern | 32 | 28 | Bloodletting therapy | Chinese herbal medicine (oral) | / | / | **IIb** |
|  | You M L（2014） | Acne | 30 | 30 | Filiform needling therapy | Western medicine (topical) | / | / |  |
|  | Qin X Y（2014） | Acne | 35 | 35 | Autohemotherapy | Western medicine (oral + topical) | / | / |  |
|  | Wu J（2014） | Acne - Lung Meridian Wind-Heat Pattern | 34 | 32 | Bloodletting therapy + Acupoint catgut embedding | Chinese herbal medicine (oral) | / | / |  |
|  | Gu C Y（2014） | Acne | 30 | 30 | Tui needling therapy + Bloodletting therapy + Pricking-cupping therapy + Filiform needling therapy | Western medicine (oral) | / | / |  |
|  | Tao X F（2014） | Acne - Spleen-Stomach Dampness-Heat Pattern | 33 | 30 | Pricking-cupping therapy + Fire needling therapy + Cupping therapy | Western medicine (oral + topical) | / | / |  |
|  | Liao J G（2017） | Acne | 31 | 32 | Fenggou needling therapy | Western medicine (topical) | / | / |  |
|  | Zuo Q Y（2018） | Acne - Lung-Stomach Dampness-Heat Pattern in Females | 30 | 30 | Bloodletting therapy | Chinese herbal medicine (oral) | / | / |  |
|  | Lu W（2018） | Acne - Post-Adolescent Females | 50 | 46 | Acupoint catgut embedding + Fire needling therapy + Auricular acupressure therapy | Western medicine (oral + topical) | / | / |  |
|  | Huang L K（2019） | Acne | 49 | 49 | Pricking-cupping therapy + Autohemotherapy | Western medicine (oral + topical) | / | / |  |
|  | Jiang J F（2019） | Acne | 30 | 30 | Fire needling therapy | Western medicine (oral) | / | / |  |
|  | Li M G（2019） | Acne | 30 | 30 | Filiform needling therapy + Acupoint catgut embedding | Western medicine (oral) | / | / |  |
|  | Yan X Z（2020） | Acne | 30 | 20 | Autohemotherapy | No treatment | / | / |  |
|  | Yan X Z（2020） | Acne | 30 | 20 | Autohemotherapy | No treatment | / | / |  |
|  | Yan X Z | Acne | 60 | 20 | Autohemotherapy | No treatment | / | / |  |
|  | Tan J Y（2022） | Acne | 30 | 30 | Filiform needling therapy + Pricking-cupping therapy | Western medicine (topical) | / | / |  |
|  | Lao Y S（2022） | Acne | 30 | 30 | Auricular acupressure therapy + Fire needling therapy | Western medicine (oral) | / | / |  |
| **Cohort study** | Cheng X Y（2014） | Acne | 64 | 60 | Filiform needling therapy + Pricking-cupping therapy | Western medicine (oral) | / | / | **IIb** |
|  | Li S S（2019） | Acne | 57 | 56 | Fire needling therapy | Western medicine (oral) | / | / |  |
|  | Xiang Y L（2020） | Acne - Cystic Type | 94 | 73 | Fire needling therapy + Acupoint catgut embedding | Western medicine (oral) | / | / |  |
|  | Wu F Q（2021） | Acne | 20 | 20 | Fire needling therapy | Debridement | / | / |  |
| **Before and after controlled trial** | Din J X（2014） | Acne - Lung Meridian Wind-Heat Pattern | 45 | / | Filiform needling therapy | / | / | / | **IV** |
|  | Zhang X P（2014） | Acne - Upper Heat and Lower Cold Pattern | 96 | / | Filiform needling therapy + Warm needling therapy | / | / | / |  |
|  | Yang S Y（2015） | Acne | 50 | / | Filiform needling therapy | / | / | / |  |
|  | Zhang Y W（2017） | Acne | 75 | / | Acupoint application therapy | / | / | / |  |
|  | Zhou L L（2019） | Acne | 16 | / | Electroacupuncture therapy | / | / | / |  |
|  | Zhou L L（2020） | Acne | 25 | / | Electroacupuncture therapy | / | / | / |  |
|  | Yan X Z（2020） | Acne | 28 | / | Autologous blood therapy | / | / | / |  |
|  | Fu Y H（2023） | Acne - Adolescent Type | 20 | / | Pricking-cupping therapy + Fire needling therapy | / | / | / |  |
| **Case series** | Jiang Y M（2014） | Acne - Adolescent Type | 50 | / | Pricking-cupping therapy + Auricular acupuncture | / | / | / | **IV** |
|  | Dong L J（2014） | Acne | 45 | / | Bloodletting therapy | / | / | / |  |
|  | Li G Q（2014） | Acne | 38 | / | Pricking-cupping therapy + Auricular acupressure | / | / | / |  |
|  | Cui X X（2014） | Acne | 39 | / | Pricking-cupping therapy + Auricular acupressure | / | / | / |  |
|  | Pang K Y（2014） | Acne - Blood Stasis and Phlegm Congestion Pattern | 60 | / | Acupoint catgut embedding | / | / | / |  |
|  | Qu X M（2014） | Acne | 42 | / | Bloodletting therapy + Gua sha therapy | / | / | / |  |
|  | Liu N Y（2014） | Acne | 31 | / | Nasal acupuncture + Bloodletting therapy | / | / | / |  |
|  | Huang Y S（2014） | Acne | 60 | / | Filiform needling therapy + Pricking-cupping therapy + Acupoint catgut embedding | / | / | / |  |
|  | Xu J R（2014） | Acne | 35 | / | Electroacupuncture therapy + Gua sha therapy | / | / | / |  |
|  | Jiao R（2014） | Acne | 36 | / | Bloodletting therapy + Auricular acupressure + Gua sha therapy | / | / | / |  |
|  | Zeng X（2014） | Acne | 50 | / | Autologous blood therapy + Gua sha therapy | / | / | / |  |
|  | Qiu B W（2014） | Acne | 46 | / | Pricking-cupping therapy + Autologous blood therapy | / | / | / |  |
|  | Guan X L（2014） | Acne | 30 | / | Filiform needling therapy + Cupping therapy | / | / | / |  |
|  | Gao Y L（2015） | Acne - Adolescent Type | 36 | / | Filiform needling therapy | / | / | / |  |
|  | Wu J M（2015） | Acne - Heat-Toxin Pattern | 37 | / | Pricking-cupping therapy + Autologous blood therapy | / | / | / |  |
|  | Qin J L（2015） | Acne | 35 | / | Filiform needling therapy + Autologous blood therapy | / | / | / |  |
|  | Yang Y C（2015） | Acne | 99 | / | Fire needling therapy + Acupoint catgut embedding + Acupoint injection + Cupping therapy | / | / | / |  |
|  | Yang D（2015） | Acne | 189 | / | Filiform needling therapy + Pricking-cupping therapy | / | / | / |  |
|  | Zhang Y J（2015） | Acne | 60 | / | Bloodletting therapy + Gua sha therapy | / | / | / |  |
|  | Zhu J H（2015） | Acne | 63 | / | Pricking-cupping therapy | / | / | / |  |
|  | Wu J Y（2016） | Acne | 30 | / | Filiform needling therapy + Pricking-cupping therapy | / | / | / |  |
|  | Liu F（2016） | Acne | 36 | / | Fire needling therapy + Cupping therapy | / | / | / |  |
|  | Fu Q F（2016） | Acne | 52 | / | Pricking-cupping therapy | / | / | / |  |
|  | Yang G J（2017） | Acne - Lung-Stomach Stagnant Heat Pattern | 48 | / | Acupoint catgut embedding | / | / | / |  |
|  | Liu Y Y（2017） | Acne - Female Type | 37 | / | Pricking-cupping therapy + Acupoint injection | / | / | / |  |
|  | Bai H H（2018） | Acne - Refractory Type | 44 | / | Pricking-cupping therapy + Autologous blood therapy | / | / | / |  |
|  | Wang Y C（2018） | Acne - Lung Meridian Stagnant Heat Pattern | 113 | / | Pricking-cupping therapy + Filiform needling therapy | / | / | / |  |
|  | Ji D D（2021） | Acne | 60 | / | Pricking-cupping therapy + Autologous blood therapy | / | / | / |  |
|  | Wang J R（2021） | Acne | 28 | / | Pricking-cupping therapy + Bloodletting therapy | / | / | / |  |
|  | Liang S（2022） | Acne | 85 | / | Filiform needling therapy | / | / | / |  |
|  | Zhang M（2023） | Acne | 32 | / | Filiform needling therapy + Pricking-cupping therapy | / | / | / |  |
| **Case report** | Li D（2014） | Acne | 2 | / | Bloodletting therapy | / | / | / | **V** |
|  | Sun H L（2015） | Acne - Adolescent Type | 1 | / | Filiform needling therapy + Pricking-cupping therapy | / | / | / |  |
|  | Liu H（2022） | Acne | 1 | / | Bloodletting therapy + Heat-sensitive moxibustion | / | / | / |  |
| **SR** | Mo Q H（2015） | Acne | 1279 | 1130 | single or combined acupuncture therapies | medicine | / | / | **Ia** |
|  | Mansu S（2018） | Acne | 1026 | | single or combined acupuncture therapies | medicine | / | / |  |
|  | Chen L L（2020） | Acne | 550 | 543 | single or combined acupuncture therapies | Western medicine | / | / |  |
